# Helicase Activity Modulation with On-Demand Light-Based Conformational Control

**DOI:** 10.1021/jacs.3c05254

**Published:** 2023-09-22

**Authors:** Dmitriy Bobrovnikov, Monika A. Makurath, Clara H. Wolfe, Yann R. Chemla, Taekjip Ha

**Affiliations:** † Department of Biophysics and Biophysical Chemistry, Johns Hopkins School of Medicine, Baltimore, Maryland 21205, United States; ‡ Department of Molecular and Integrative Physiology, University of Illinois at Urbana-Champaign, Urbana, Illinois 61801, United States; § Department of Physics, University of Illinois at Urbana-Champaign, Urbana, Illinois 61801, United States; ∥ Center for Biophysics and Quantitative Biology, University of Illinois at Urbana-Champaign, Urbana, Illinois 61801, United States; ⊥ Department of Biophysics, Department of Biological Engineering, Johns Hopkins University, Baltimore, Maryland 21218, United States; # Howard Hughes Medical Institute, Baltimore, Maryland 21205, United States

## Abstract

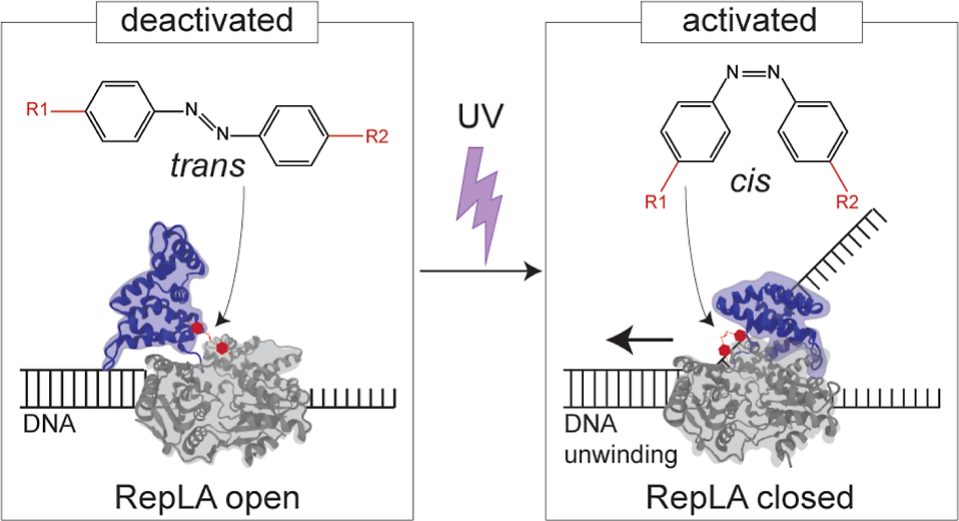

Engineering a protein variant with a desired role relies on deep knowledge of the relationship between a protein's native structure and function. Using our structural understanding of a regulatory subdomain found in a family of DNA helicases, we engineered novel helicases for which the subdomain orientation is designed to switch between unwinding-inactive and -active conformations upon *trans–cis* isomerization of an azobenzene-based crosslinker. This on-demand light-based conformational control directly alters helicase activity as demonstrated by both bulk phase experiments and single-molecule optical tweezers analysis of one of the engineered helicases. The “opto-helicase” may be useful in future applications that require spatiotemporal control of DNA hybridization states.

## Introduction

DNA is an instruction manual, efficiently encoding information needed for gene expression and protein synthesis. Additionally, its structural characteristics as a long, flexible, polymeric molecule that is programmable, able to form secondary structures and to bind proteins and other nucleic acids, lend themselves well to many technological and biological applications. For example, custom DNA origami shapes can be synthesized based on the complementarity of nucleobases,^1^ and the physical characteristics of DNA ensure its ability to be precisely packed, read, and replicated. While we know how to design and apply DNA nanostructures and how the DNA duplex is unwound to initiate biological processes such as transcription, replication, and repair, our ability to control the hybridization state of the DNA duplex at high spatiotemporal resolution is still limited. The ability to use nucleic-acid processing enzymes in a controlled manner to manipulate DNA structures at predetermined locations and time points would thus be highly valuable.

Helicases are a broad class of enzymes that are responsible for processing nucleic acids in all domains of life and play important roles in DNA replication, recombination, and repair, among other vital nucleic acid metabolism processes.^2−6^ To carry out their functions, especially those requiring directional motion, helicases consume chemical energy from nucleoside triphosphates, most commonly adenosine triphosphate (ATP), and turn it into mechanical energy allowing them to move along strands of a nucleic acid. This movement allows helicases to displace nucleic-acid-bound proteins,^7^ resolve DNA secondary structures,^8^ and dissolve the double helix.^7^ However, their ability to do so is context-dependent, requiring fine-tuned adaptation to specific conformations, oligomerization states, or stimulation by other external factors.^7,9^

**Figure 1:**
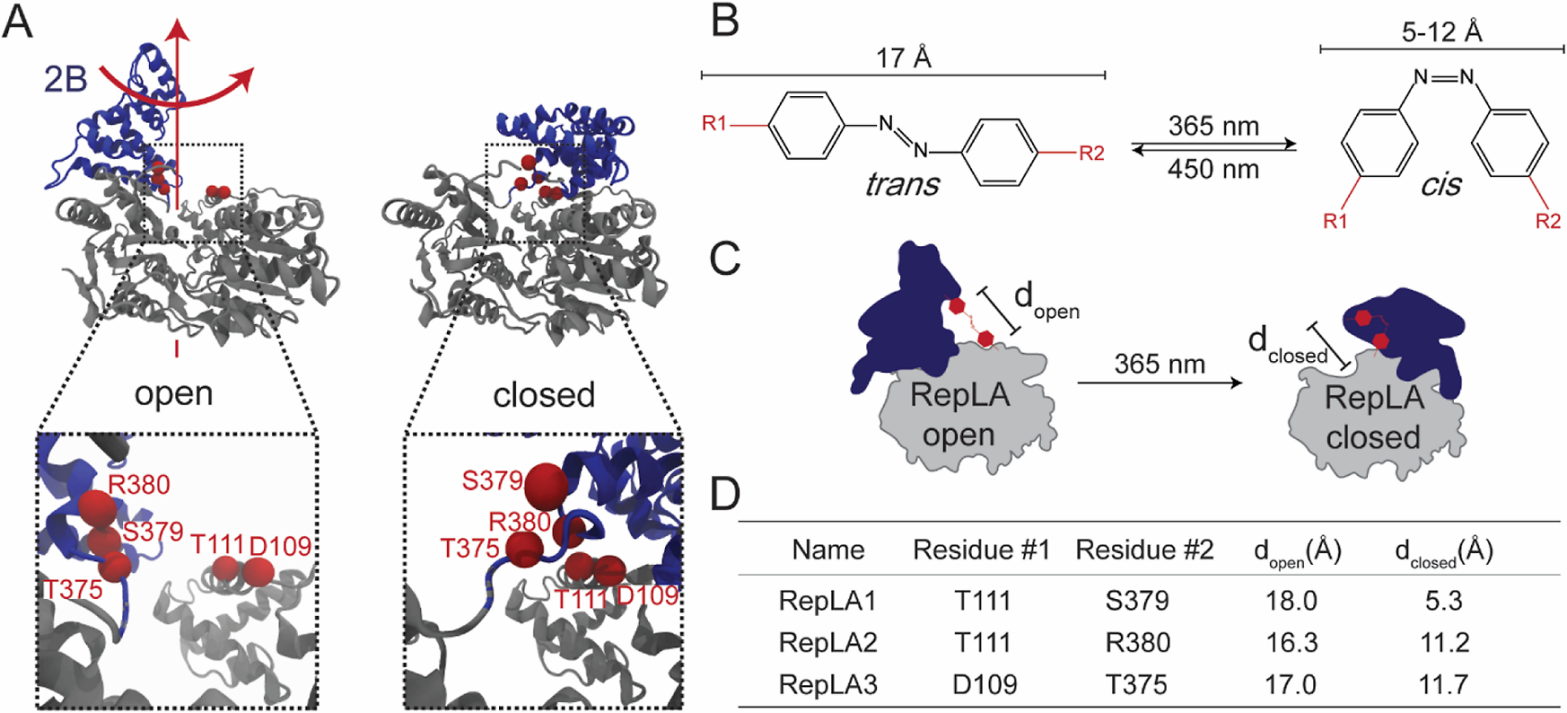
(A) Crystal structure of Rep helicase in two conformations, open (left) and closed (right), based on the rotation of the 2B subdomain. The crystal structures of both open and closed conformation are from PDB 1UAA(10) and are rendered using visual molecular dynamics. The red arrow highlights rotation of the 2B subdomain (blue) relative to the subdomains 1B, 1A, and 2A (gray). Insets highlight the residues (red) mutated for azobenzene incorporation in the open (left inset) and closed (right inset) conformations. (B) Photoisomerization of azobenzene. Azobenzene transitions from *trans* (left) to *cis* (right) state when illuminated with 365 nm light. The reaction is reversible by illumination with 450 nm light or heat. (C) Schematic depicting the design strategy for the RepLA proteins. (D) Residue pairs for incorporation of azobenzene into RepLA proteins and corresponding distances between the selected residues in the open (*d*_open_) and closed (*d*_closed_) conformations calculated from the crystal structure of Rep.

Of the large structural superfamily 1 (SF1) of DNA helicases, the three most extensively studied proteins are Rep, UvrD, and PcrA, which exhibit high sequence and structural homology.^10−12^ These helicases have been shown to unwind DNA efficiently as dimers,^13^ although monomer unwinding can be promoted through destabilizing the duplex by force,^14^ interactions with protein partners,^15^ or conformational control of a regulatory subdomain, 2B.^16−18^ This structural subdomain can adopt multiple orientations,^10−12,19^ the extremes of which correspond to conformations known as “open” and “closed” (Figure 1A), that are coupled to their protein displacement^20^ and unwinding activities.^12,21^ Early studies demonstrated an auto-inhibitory role for 2B in Rep, where 2B removal to generate the RepΔ2B variant increased the extent of helicase unwinding as measured by higher processivity and an increased unwinding speed on double-stranded (ds) DNA, accompanied by an increase in the speed of translocation on single-stranded (ss) DNA.^14,17^ Single-molecule experiments have further shown that a single UvrD molecule adopts the “closed” conformation during duplex unwinding.^21^ The transition from the “closed” to “open” conformation correlates with a “strand-switching” behavior where the helicase loads on the opposite strand of the helix and moves in the opposite direction to unwinding, allowing the DNA to rezip.^21,22^ Additional single-molecule experiments have revealed that permanent site-specific chemical crosslinking of Rep into the “closed” conformation (Rep-X) creates a “superhelicase” that is capable of highly processive unwinding of long stretches (>6 kb) of duplex DNA as a monomer.^16^ Although Rep-X is an SF1 variant that is the most successful at uninterrupted unwinding of long stretches of DNA, it requires a free 3′-tail to load onto the DNA, and its direction of motion (the ability to switch-strands) cannot be controlled. Crosslinked superhelicases based on Rep and PcrA have been used for mimicking cotranscriptional RNA folding,^23,24^ genome imaging via local denaturing,^25,26^ remodeling of small catalytic DNA hairpins,^27^ and isothermal DNA amplification.^28^ The conformation of the 2B domain of Rep is clearly important for its enzymatic function and controlling this conformation has led to the development of several molecular tools. The ability to control the 2B domain in a precise and reversible manner would enable further study of the association between Rep structure and function and may be applied to novel technologies.

Here, we created light-activatable variants of Rep helicase, RepLAs, where we aimed to control in real time the 2B subdomain orientation (Figure 1A) with an external light source. To achieve this goal, we engineered Rep with a bifunctional cysteine-reactive crosslinker containing an azobenzene moiety that is capable of isomerizing from *trans* to *cis* states upon exposure to UV (365 nm) light (Figure 1B). This azobenzene crosslinker is used to covalently join two cysteines that have been specifically site-engineered to modulate the 2B subdomain orientation through direct control of the distance separating them (Figures 1C and S1A–D).

## Materials and Methods

### RepLA Design

To design light-activatable versions of Rep helicase (RepLA, Figure 1C), we mutated pairs of residues to cysteines in an otherwise cysteine-free Rep mutant such that when crosslinked, the distance between residue positions in the “open” and “closed” conformations corresponds to the *trans* and *cis* isomer lengths of azobenzene (Figure 1B–D). We selected the residues by distance constraints as well as by solvent accessibility^29−31^ (Figures 1D, S1, and Supporting Information text). Our selection of residues was assisted by Chimera^32^ which allowed us to replace each single amino acid in both the open and closed conformations of Rep in the crystallographic structure^10^ (Protein Data Bank entry 1UAA) with cysteines. Replacements were made using the cysteine rotamer that most closely approximated the conformation of the native residue. We calculated the accessible surface area for each mutated residue^30,33^ as well as the distance between the estimated gamma atom coordinates for every mutated pair of cysteines in each conformation (Figure S1C). We selected the residue pairs that fit the criteria for the distance constraints of *cis* and *trans* azobenzene and maximized the solvent accessible surface area of the least-accessible member of each “open”/”“closed” pair considering both conformations that could be present during the chemical crosslinking (Figure S1D). Manual inspection of each candidate cysteine pair minimized the likelihood that the selected residues had a functional role in ATP or DNA binding.^10,34,35^

### Cloning, Expression, and Purification

To make the different versions of RepLA, we cloned a cysteine-free mutant of Rep helicase containing a 6xHis tag as described previously^16,34^ in a pET28a vector. This cysteine-free template was then used to insert the desired cysteines selected for crosslinking in two rounds of single-cysteine insertion using site-directed mutagenesis using mutagenic oligonucleotide primers and the Q5 polymerase chain reaction (PCR) Kit (NEB). Cloning was performed with DH5α *Escherichia coli* cells. Cloned and Sanger-sequencing-confirmed plasmids were then moved to BL21 *E. coli* cells where proteins were expressed with IPTG induction and purified with a Ni-NTA column as done previously^34^ and confirmed by sodium dodecyl sulfate-polyacrylamide gel electrophoresis (SDS-PAGE) for purity. Proteins were stored in Buffer B: 50 mM TrisHCl (pH 7.6 at room temperature), 1 mM ethylenediaminetetraacetic acid (EDTA), 600 mM NaCl, and appropriate amounts of glycerol (25–50%) when storing at −80 °C.

### Crosslinking RepLA Proteins

**Figure 2:**
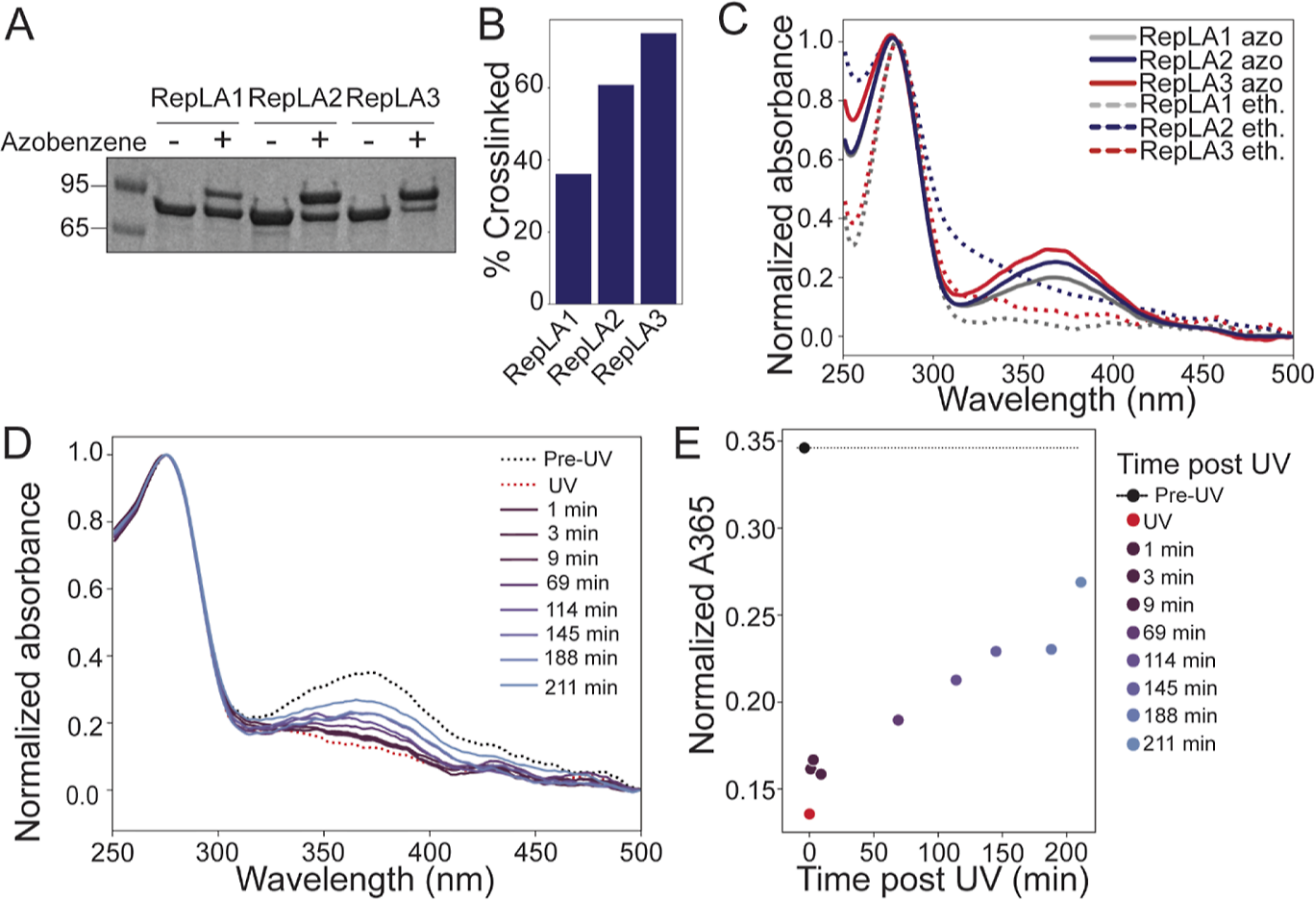
(A) SDS-PAGE analysis of the purified RepLA proteins pre-(−lane) and post-(+lane) incubation with bismaleimide-azobenzene reveals the appearance of a shifted band arising from an intramolecularly crosslinked population of protein. (B) Crosslinking efficiency of the RepLA proteins quantified from (A). (C) Incorporation of an azobenzene crosslinker can be additionally confirmed by the presence of an absorbance peak at 365 nm (solid lines) which is not observed in proteins crosslinked with an ethane linker (dotted lines). (D) Time series of normalized UV–vis absorbance spectra of RepLA2 starting immediately after a 2 min 365 nm irradiation. Each trace represents a single time point after UV irradiation. Each UV–vis trace is normalized using min–max normalization based on the aromatic absorbance peak at 280 nm and the calculated minimum absorbance (500 nm). The equilibrium *trans* state of the azobenzene in RepLA2 is shown with the spectra denoted by the dotted black line while the measurement immediately following UV treatment is depicted by a dotted red line. (E) Plotting normalized absorbance at 365 nm vs time reveals the kinetics of the transition of the protein-incorporated azobenzene from its high-energy *cis* state to the lower energy *trans* state under dark, room-temperature conditions. The absorbance of the equilibrium *trans* state is depicted by a black point and dotted black line. The absorbance measurement taken immediately after UV illumination is depicted by a red point. The time series reveals that the isomerization for RepLA2-incorporated azobenzene from *cis* back to *trans* in the dark at room temperature, after 2 min illumination with UV light, occurs with a half-life of approximately 175 min.

Proteins were crosslinked with either 4,4′-bis(maleoylamino)azobenzene (Toronto Research Chemicals) or bismaleimioethane (Figure S1B) dissolved in dimethylformamide (DMF). Crosslinker solution was added to eluted protein solution for a final ratio of 4.4:1 crosslinker/protein such that the final solution contained <2% DMF. The reaction was rotated at room temperature in the dark for 1.5 h, spun down to remove precipitated crosslinkers, and dialyzed into Buffer B at 4 °C overnight. Crosslinking was confirmed by a denaturing SDS-PAGE gel (Figures 2A and S2) that displays a characteristic band retardation of the protein for the crosslinked fraction due to chemical crosslinking between residues that are sufficiently separated in the primary protein sequence. A secondary confirmation was performed for all proteins using UV–vis spectroscopy where a characteristic absorbance peak at 365 nm (A365) is expected for the incorporated azobenzene moiety in its *trans* state (Figure 2C).

### UV–Vis Spectroscopy

UV–vis spectroscopy was used to confirm the incorporation of azobenzene into crosslinked protein samples as well as monitor the isomerization state of samples after exposure to either blue light or UV light. All UV–vis experiments were performed using a Nanodrop 2000 instrument that was previously blanked with protein storage buffer B. Protein spectra were measured at room temperature. Spectra were smoothed using a rolling window of 20 nm and normalized using min–max normalization with the aromatic peak at 280 nm set to 1 and the calculated minimum absorbance lying between 250 and 500 nm set to 0. For isomerization testing, spectra from fresh aliquots of protein were measured before any light exposure. Samples were then exposed to light in thin-walled PCR tubes as described, and spectra were measured again. For temporal isomerization experiments, approximations for half-life of isomerization were calculated using the linear regression of normalized 365 nm absorbance (not shown) and determining the time point at which the A365 reached the calculated midpoint between the A365 of the *cis* and *trans* states.

### Helicase Activity Testing

Helicase activity assays performed in bulk utilized an 18 bp dsDNA duplex consisting of an 18 nt DNA strand labeled with a fluorescein at the 5′ end and a dabcyl quencher on the 3′ end and hybridized to a 28 nt strand containing a (dT)_10_ tail to allow for helicase loading. All protein samples were pretreated with 455 nm light (M455L4-C1 ThorLabs) in thin-walled PCR tubes for 1 min prior to addition to the reaction mixture to ensure a consistent *trans* isomerization state. UV-treated samples were irradiated for 30s with 365 nm light (uvBeast 365 nm mini flashlight) in thin-walled PCR tubes prior to addition to the reaction buffer. Reactions were initiated by adding the substrate DNA alongside adenosine triphosphate (ATP) to solutions containing the protein. All experiments were performed at room temperature in the dark. The final reaction contained 20 nM protein, 50 nM substrate DNA, 1 mM ATP, 10 mM MgCl_2_, 100 mM TrisHCl (pH 8.0), 10% glycerol, and 15 mM NaCl. Reactions were quenched after 10 min by the addition of an equal volume of twofold concentrated quenching buffer containing 20 mM EDTA, 0.5% SDS, and 250 nM of an unlabeled single-stranded 18 nt reporter DNA in the final-quenched volume. Final fluorescence was measured 10 min after quenching with a Qubit 3.0 fluorometer using 480 nm excitation and emission read at 510–580 nm. Fluorescence was reported in relative fluorescence units (RFUs) using the default values of the Qubit instrument. All values depicted here were normalized to a control condition containing all reaction components except ATP (Figure S4A,C), which, as expected, showed the highest fluorescence for each experiment to allow for better comparison across conditions. For assays performed under reducing conditions, reaction buffer was prepared as above with the addition of 100 mM dithiothreitol (DTT). Proteins were exposed to light as described above, added to the appropriate amount of reaction buffer, and then incubated for 10 min at room temperature in the dark. The protein and reaction buffer were then combined with appropriate amounts of DNA reporter and ATP, and the assay was quenched and fluorescence was measured as described above. DNA constructs are included in Table S1.

### Single-Molecule Measurements

#### DNA Hairpin Construct

We carried out all single-molecule experiments on a DNA hairpin construct as described previously.^21^ Briefly, the construct consists of an 89 bp hairpin stem capped by a (dT)_4_ loop (allowing to measure unwinding of up to 91 bps) and contains at its 3′ end a 10 dT single-stranded DNA region that serves as a Rep helicase loading site (Figure 4A, left). The 10 nt length of the ssDNA tail can accommodate only a single Rep molecule.^13^ The hairpin ends were ligated to 1.5 kb dsDNA handles each modified with a single biotin or digoxigenin for attachment to streptavidin- and anti-digoxigenin antibody-coated beads, respectively. Sequences for all hairpin inserts and primers are displayed in Table S1, and additional details are described in Supporting Information text.

#### Optical Trapping Instrumentation

We modified a dual-trap optical tweezers instrument^36^ to provide epi-illumination of UV light at the sample plane. We configured a 365 nm LED (M354 LP1C1, ThorLabs) and collimated the light beam prior to introducing UV into the path of the optical trap via a beam-splitting cube (Figure S7). We focused the light at the back focal plane of the front objective to ensure epi-illumination of the specimen plane (Figure S7A,D). The UV power was kept low within a range of ∼1–10 mW to avoid possible photodamage to the sample. We controlled the UV light through a computer interface. When noted, we tracked the timing of UV illumination (Supporting Information text). We acquired all optical trap data at 100 Hz.

#### Sample Chamber and Unwinding Assay

Measurements were carried out in a microfluidic sample chamber^37^ consisting of Nescofilm mask (Karlan, Phoenix, AZ) patterned with three flow channels and adhered to two microscope coverslips (Figure S8). The coverslips were passivated with polyethylene glycol to reduce protein adsorption. The outer channels were connected to the central channel via glass capillaries (Garner Glass Co., Claremont, CA) embedded in the Nescofilm. The top and bottom channels contained DNA-coated streptavidin- and anti-digoxigenin antibody beads, respectively, that flowed into the central channel where they could be captured by the optical traps. The central channel contained two adjacent laminar flow streams composed of different buffers that did not mix significantly. One stream contained ATP (250 μM) but not Rep helicase, while the other contained Rep helicase (25 nM) but not ATP.

#### Optical Trapping Experimental Conditions

We performed our experiments in the dark (unless otherwise noted), at 22 °C, in 35 mM Tris–HCl (pH 8.0), 20 mM NaCl, 5 mM MgCl_2_, and 2% glycerol. The media contained an oxygen-scavenging system consisting of 1.2% glucose, 0.29 mg/mL pyranose oxidase from *Coriolus* sp. (Sigma-Aldrich, St. Louis, MO, USA), and 0.15 mg/mL catalase from *Apergillus niger* (EMD Millipore, Billerica, MA, USA) to increase the lifetime of the DNA tethers and reduce photodamage to the sample.^38^ Unless otherwise noted, we used 25 nM Rep and 250 μM ATP for all measurements.

#### Continuous and Discontinuous UV Illumination

All experiments were done under single-turnover conditions, where a single helicase was allowed to bind and unwind the DNA hairpin before dissociating (Figure S8). First, a single-DNA hairpin was tethered between two optically trapped beads in the ATP stream. The tether and beads were then translated into the protein stream, allowing a single Rep molecule to bind to the 10 nt loading site but not to unwind DNA due to the absence of ATP. Finally, the complex was returned to the ATP stream where unwinding could initiate. An active feedback loop maintained a constant tension of 11 pN throughout. In measurements with continuous UV illumination (Figures 4 and S8A), the UV light source was turned on during all the steps described above, i.e., when loading Rep onto the DNA in the protein stream and when unwinding in the ATP stream. For experiments performed under discontinuous UV illumination (Figures S8B and S9), UV illumination was only initiated once the DNA with bound protein had entered the ATP channel. The details of each protocol are described in the Supporting Information Text.

#### Data Analysis

The amount of DNA unwound (in units of base pairs) at each time point was calculated by dividing the change in tether extension (in nm) by the extension of the two nucleotides of ssDNA released per unwound base pair at the measured force. The latter was determined from the extensible worm-like chain (XWLC) model for ssDNA using the following parameters:^21^ persistence length, *P*_ss_ = 1 nm, inter-phosphate distance, *h*_ss_ = 0.59 nm/nt, and stretch modulus *S*_ss_ = 1000 pN. These XWLC model parameters, along with those for dsDNA (*P*_ds_ = 53 nm, *h*_ds_ = 0.34 nm/bp, and *S*_ds_ = 1100 pN), were verified by fitting the force-extension behavior of a single molecule of the DNA hairpin construct obtained from mechanical unfolding and refolding by the optical traps, as described previously.^21^ The measurement of RepLA2 activation time is described in Supporting Information text.

## Results

### Rep Crosslinked with Photoactive Azobenzene

To create light-activatable variants of Rep helicase, we exploited the ability of a bismaleimide-azobenzene crosslinker, henceforth referred to as azobenzene, to change the distance between two cysteines to which it is crosslinked^29,31,39^ (Figure 1B). The cysteine locations were rationally chosen based on the known crystal structure of Rep helicase in its two different conformations^10^ (Figure 1A) and our knowledge of the function arising from the two conformations.^16,21^

We set out to design crosslinked variants of Rep that under the *trans* azobenzene state, populated in the absence of UV light, would be in the unwinding-inactive “open” conformation and under the *cis* azobenzene state would be in the unwinding-active “closed” conformation (Figure 1C). We named these constructs RepLA for “Light-Activated”. To find the appropriate pair of residues to mutate into cysteines, we required the distance between the side-chain gamma atoms to be approximately 17 Å for the “open” conformation, corresponding to the para–para distance of *trans* azobenzene including both maleimide crosslinkers^29,31^ and approximately 5–12 Å for the “closed” conformation, corresponding to the para–para distance for functionalized *cis* azobenzene. Briefly, we replaced, in silico,^32^ each residue the Rep “open” and “closed” crystal structures, with a cysteine using the most similar rotamer conformation to the native mutated residue. The distances between the gamma sulfur atoms of each newly inserted pair of cysteines were calculated. After selecting residue pairs with the inter-residue distances in each helicase conformation, we further filtered candidate residues based on their solvent accessibility to ease crosslinking (Figure S1 and Supporting Information Text). We excluded by visual inspection residues likely to play an important role in ATP or DNA binding. We picked three residue pairs that satisfied our criteria (Figure 1D) and synthesized the corresponding protein variants, named RepLA1, RepLA2, and RepLA3.

Following the cloning and purification of all three of the RepLA proteins, a crosslinking reaction was performed with the azobenzene crosslinker, and the proteins were subsequently analyzed by SDS-PAGE to determine the extent of crosslinking (Figures 2A,B and S2) and by UV–vis spectroscopy to ensure the incorporation of the azobenzene linker (Figure 2C). If the two crosslinked cysteines are sufficiently separated in the protein sequence, we observe a clearly shifted band for an intramolecularly crosslinked protein compared to uncrosslinked protein in SDS-PAGE analysis (Figure 2A), allowing for not only the confirmation of crosslinking but also the ability to measure the extent of crosslinking (Figure 2B). Small amounts of higher order intermolecular crosslinking occasionally occur during crosslinking, but these structures may not be active and represent a negligible amount of protein at the concentrations used for the assays described here (Figure S2). The gel-band intensities (Figure 2B) indicate that the RepLA proteins are 35–60% crosslinked with minor variability observed in independent crosslinking reactions (Figure S2).

To confirm the incorporation of the azobenzene crosslinker, we used the characteristic absorbance of *trans*-state azobenzene which displays a peak between 316 and 400 nm depending on solvent and local environment.^29,40,41^*Cis* azobenzene does not display this characteristic peak, and the isomerization state of azobenzene can, therefore, be monitored spectroscopically. All three RepLA proteins exhibit a local absorbance maximum at 365 nm (Figure 2C) which is not seen in proteins that are crosslinked with a bismaleimideoethane (ethane) crosslinker (Figure 2C) or uncrosslinked proteins (Figure S3A).

Azobenzene undergoes photoisomerization from *trans* to *cis* upon UV (365 nm) irradiation and returns to the *trans* state either spontaneously or upon blue (455 nm) light irradiation.^40^ To characterize the isomerization of the incorporated azobenzene, we next performed a temporal study of isomerization after UV irradiation in one of the proteins, RepLA2, which displayed the largest light dependence in a bulk unwinding assay. The UV–vis spectra were measured before UV (365 nm) irradiation to establish the profile for the *trans* state of azobenzene, and then the sample was irradiated for 2 min with UV light to convert azobenzene into the *cis* state. The UV–vis spectra were then measured immediately after UV irradiation as well as at various time points over several hours, while the protein was held in the dark at room temperature, allowing azobenzene to relax to the *trans* state. Upon exposure to UV, the absorbance at 365 nm decreased, indicating conversion to the *cis* state (Figure 2D). The temporal relaxation back to the *trans* state can also be seen by the return of the 365 nm peak to its pre-UV irradiation state. This experiment demonstrates that under these conditions the protein-incorporated azobenzene can be switched to the *cis* state, which then relaxes to the *trans* state with a half-life on the order of hours (Figure 2E), in agreement with values in the literature on the order of 120 min for protein-bound azobenzenes.^42^ To demonstrate that azobenzene crosslinked proteins remain predominantly in the *trans* conformation during and after crosslinking, we irradiated RepLA2 with several different time exposures to 455 nm light which should induce the *trans* isomer of azobenzene. We saw no difference in the 365 nm absorbance peak between unmodified protein in the equilibrium state and protein exposed to blue light (Figure S3B). We additionally tested the isomerization kinetics of RepLA2 with consistent exposure to ambient room light after UV exposure (Figure S4). Samples that were exposed to ambient light exhibited more rapid isomerization to the *trans* state which is consistent with greater exposure to blue wavelengths of light than ultraviolet wavelengths of light from ambient white light. Subsequent experiments were, therefore, performed in the dark and can be assumed to occur in the *cis* state when UV-treated due to its long-lasting nature.

### Light-Dependent Unwinding Activity in Bulk Solution

**Figure 3:**
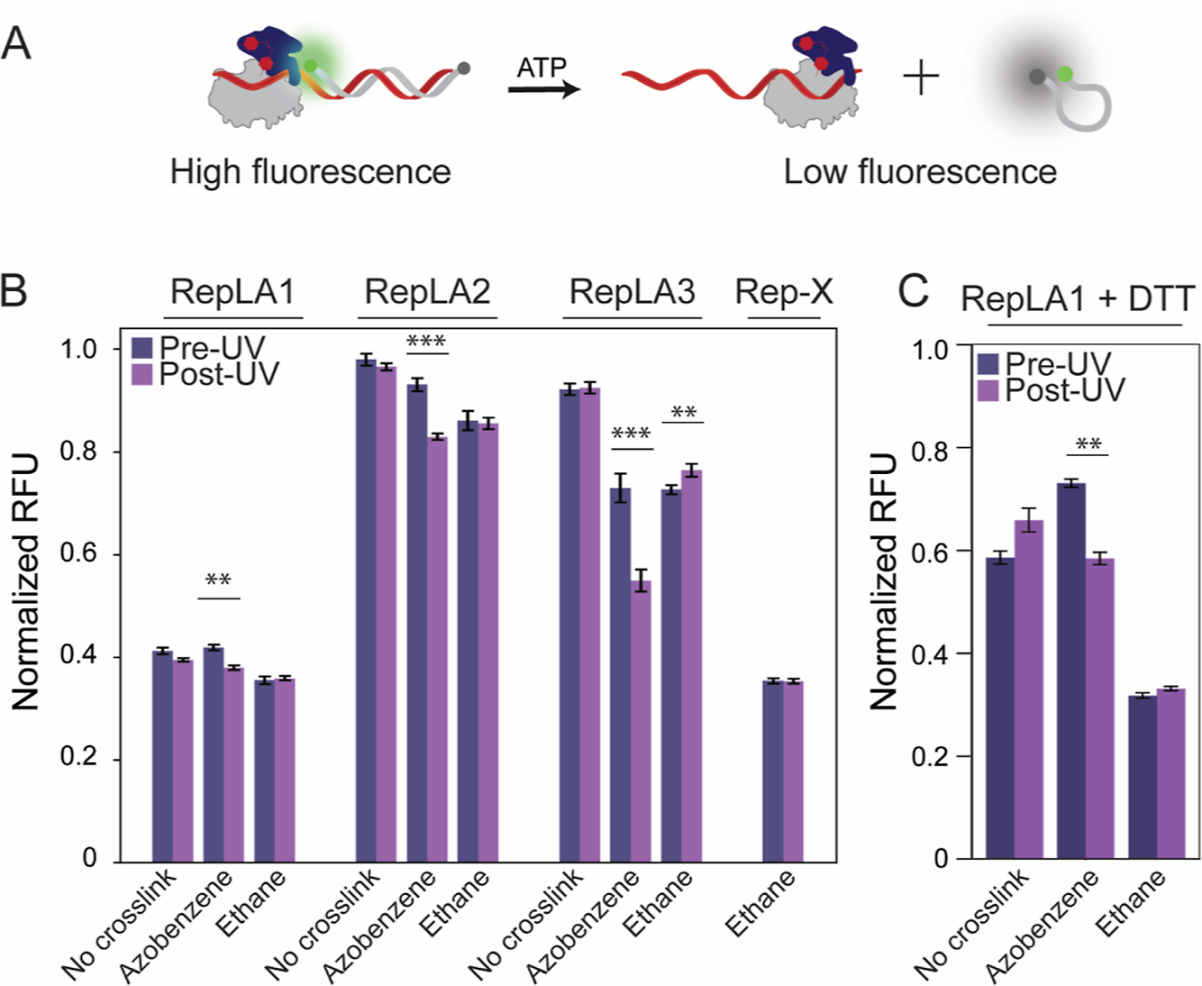
(A) The bulk dsDNA unwinding assay is initiated by a rapid combination and mixing of DNA, ATP, and helicase and allowed to proceed for 10 min before quenching with SDS and an excess of unlabeled DNA strand. The 5′ fluorescein-labeled 18-mer DNA exhibits a high level of fluorescence when hybridized to another strand, and the fluorescence is quenched when the single strand is released by the helicase due to increased proximity between fluorescein and the 3′ dabcyl quencher. (B) Bulk unwinding assay performed using RepLAs. For each RepLA design, proteins remained in their native uncrosslinked states or were crosslinked with an azobenzene or an ethane crosslinker. All unwinding reactions were normalized to a negative control condition containing ethane crosslinked proteins and no ATP, showing the highest fluorescence for each measurement window. We note that a minimum fluorescent value was not taken into account when normalizing. The ethane crosslinker mimics the *cis*-state azobenzene crosslinker and thus exhibits increased unwinding in all samples. Helicase activation in response to pre-treatment with 30 s UV light is significant in all samples. Paired *t*-tests were used to calculate the significance of the differences between untreated (*n* = 8, blue bars) and UV-treated samples (*n* = 8, purple bars) for each condition. Data are displayed as mean and standard error. ***p < 0.001, **p < 0.01, *p < 0.05. Differences between UV and untreated conditions for each sample with *p* > 0.05 are not marked. (C) RepLA1 unwinding in the presence of 100 mM DTT. Treatment with a reducing agent partially abrogated the high baseline unwinding exhibited in the uncrosslinked condition and also decreased background unwinding in the azobenzene crosslinked condition (*n* = 4 for each condition), indicating that much of the background unwinding activity results from oxidation of the unreacted cysteines in the protein. Data are displayed as mean and standard error. **p < 0.01. Differences between UV and untreated conditions for each sample with *p* > 0.05 are not marked.

To characterize the unwinding activity of the RepLA helicases in bulk solution, we developed a fluorescence-based DNA unwinding assay. The unwinding substrate contained 18 bp dsDNA with a 3′ (dT)_10_ overhang for helicase loading (Table S1). The strand complementary to the loading strand was labeled with a fluorescein on the 5′-end and a fluorescence quencher on the 3′-end. When RepLA unwinds this substrate and displaces the labeled strand, the released single strand will coil because of its high flexibility,^43^ and the fluorophore will be quenched (Figure 3A). We previously showed that crosslinking two residues within Rep to constitutively maintain the 2B “closed” conformation produces a superhelicase, Rep-X.^16^ We used unwinding of the reporter by Rep-X to validate our assay, showing that a decrease in fluorescence intensity, measured by RFUs or RFU, acts as a readout for the extent of unwinding, as demonstrated by comparing reactions in the absence and presence of ATP (Figure S4A) and at increasing concentrations of helicase (Figure S4B).

We posited that the RepLA proteins would exhibit negligible unwinding when they are left in their native, uncrosslinked state, as the short 10 nt overhang on the dsDNA reporter would not accommodate dimers of Rep, which are necessary for unwinding activity in its native state.^13^ As expected, at the protein concentrations tested, uncrosslinked RepLA2 and RepLA3 do not unwind any significant amount of duplex (Figure 3B). Interestingly, RepLA1 exhibits some amount of unwinding without crosslinking, nearly equivalent to the unwinding capacity of a statically linked RepLA1 helicase. We attribute this result to partial oxidation of the two cysteines into disulfides in some population of the helicases, as we have shown in the case of PcrA-X.^16,28^ We note that the distance between two cysteines in the closed conformation is much smaller for RepLA1 compared to that for RepLA2 or LA3 (Figure 1D), consistent with a higher probability of disulfide bond formation. Disulfide bonds may also be responsible for our observation that RepLA1 has a lower crosslinking efficiency as quantified by gel shift on a reducing gel (Figure 2B), as disulfide bond formation would compete with the azobenzene crosslinking reaction. As such, we performed the same unwinding assay with RepLA1 which was incubated with 100 mM DTT for 10 min prior to initiation of unwinding and for the duration of the assay (Figure 3C.). The DTT treatment partially abrogated the high background unwinding seen in the uncrosslinked samples, as well as increased the dynamic range in the azobenzene crosslinked samples, indicating that disulfide formation is at least partially responsible for the increased background unwinding. DTT treatment had a markedly smaller effect on RepLA2 and RepLA3 (Figure S5D), possibly because the greater distance between the crosslinked cysteines in these constructs minimizes disulfide formation in solution.

As a positive control, we tested whether RepLAs would exhibit enhanced unwinding when crosslinked with the same ethane crosslinker utilized for Rep-X. All three helicase constructs, when crosslinked with the ethane crosslinker, exhibit the expected increase in unwinding capacity as measured by the decrease in fluorescence intensity relative to the sample without helicase. The drop in fluorescence intensity in the two-carbon crosslinked proteins compared to that of the uncrosslinked helicases demonstrates that the ethane crosslinker is indeed capable of enhancing the unwinding activity (Figure 3B). Variations in unwinding efficiency between different constructs crosslinked with the ethane moiety most likely arise from the differences in the distances and orientations of the crosslinked residues, the positions of which were optimized for the azobenzene crosslinker. Notably, RepLA1 has an inter-cysteine distance of 5.3 Å, which is shorter than the ethane linker length of roughly 8 Å which may allow more efficient crosslinking by ethane, while the distance between cysteines in RepLA2 and RepLA3 is larger and may prevent efficient crosslinking by the ethane linker. This may explain why RepLA2 and RepLA3 exhibit lower overall activity when crosslinked with an ethane crosslinker than do RepLA1 and Rep-X.

To determine whether the azobenzene crosslinker enables dynamic control of helicase activity, we measured the activity of the RepLA proteins crosslinked with before and after UV exposure. All proteins were treated with of 455 nm light exposure to ensure consistent *trans* state occupancy prior to the start of the experiment. Helicases were activated separately through 30 s illumination with a 365 nm light before being added to the reaction mix to ensure that UV light acted on the proteins alone. Reactions were initiated with the addition of substrate DNA and ATP. All three azobenzene-containing RepLA proteins exhibit a significant enhancement of their unwinding when pre-activated with UV light (Figure 3B). For comparison, none of the RepLA constructs crosslinked with the ethane crosslinker showed unwinding enhancement when pre-treated with UV light (Figure 3B), nor did Rep-X.

Based on the findings of the bulk assay which showed that RepLA2 crosslinked with azobenzene had a significant UV response with the lowest background activity when not exposed to UV light, we chose to further characterize the isomerization and unwinding dynamics of this construct.

### Dual-Optical Trap with UV Illumination

Next, we employed a DNA hairpin unwinding assay using optical tweezers^14,21,44^ to examine, at the single-molecule level, RepLA2. A DNA hairpin, which allows measurement of processivity up to 91 bp, was tethered between two microspheres each held in traps. The distance between the traps was adjusted using a feedback system such that the DNA molecule was held under a constant tension of 11 pN. All experiments were carried out in sample chambers consisting of two buffer channels, one channel with 25 nM protein and the other channel with 250 μM ATP (Figure S8). Placing the tethered hairpin into the protein channel allowed RepLA2 to bind to the 10 nt ssDNA site, which can accommodate only a monomer of the protein.^13^ Unwinding was then initiated by moving the tethered hairpin with bound RepLA2 into the ATP channel. Experiments were performed with and without UV exposure (SI text). Light activation was carried out in situ with epi-illumination of the sample chamber with 365 nm light from an LED (Figure S7). The UV light was continuously on during RepLA2 loading in the protein channel and during unwinding in the ATP channel (Figure S8A). The UV power at the specimen plane was kept at 1–10 mW.

**Figure 4:**
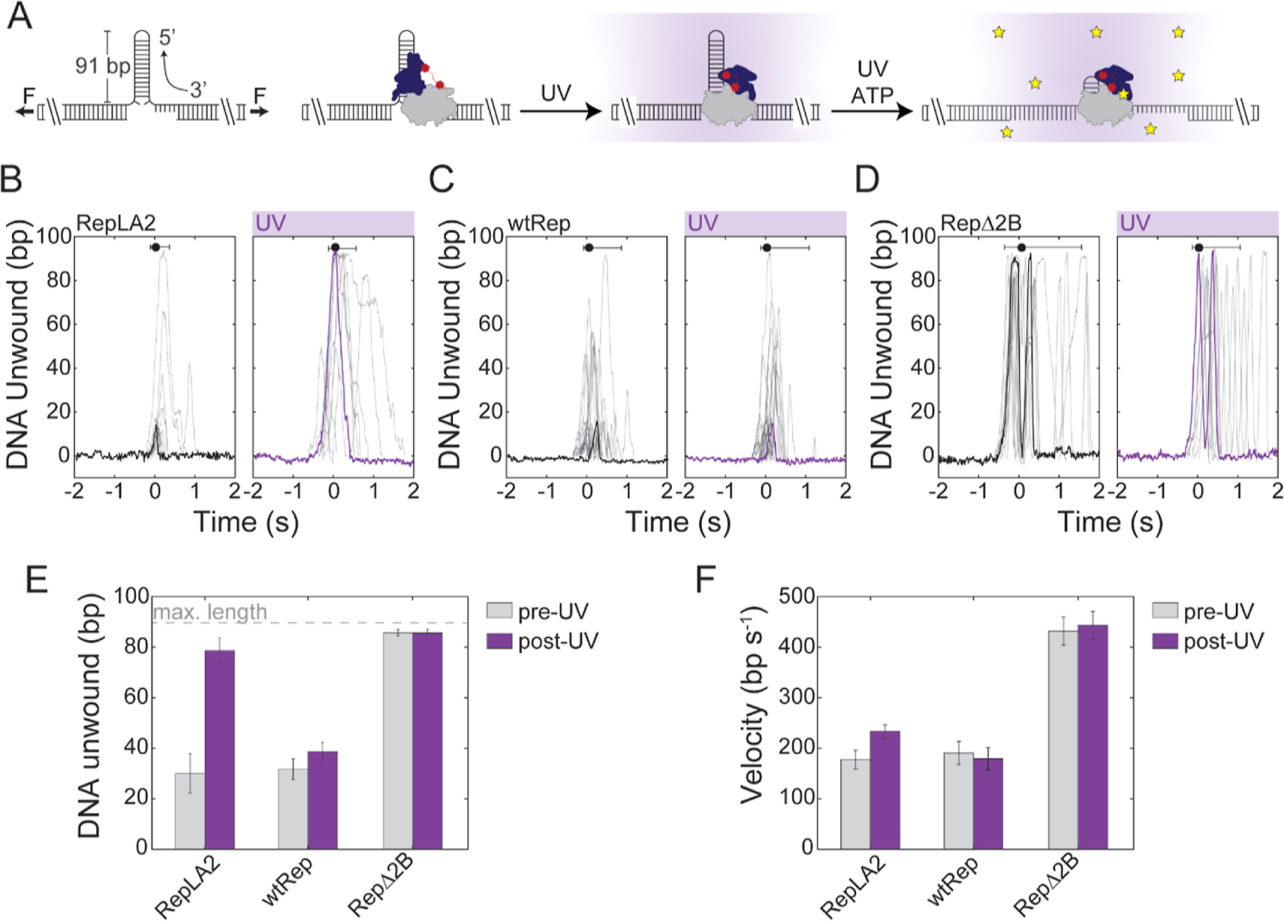
(A) Schematic illustration of the continuous in situ UV illumination protocol during a single-molecule optical trapping experiment. A DNA hairpin is held under force by optical traps. A monomer of Rep or variant binds to an ssDNA-loading site 3′ of the hairpin; unwinding is initiated by the addition of ATP (yellow stars). Protein loading and unwinding happen in the presence of UV light. The UV (365 nm LED) power at the specimen plane is ∼1–10 mW. (B) Representative traces of RepLA2 without UV (left, *n* = 11) and with UV (right, *n* = 11). (B–D) Traces are aligned to start at *t* = 0, calculated as the time at which the trapped DNA construct reaches the midpoint between the protein and ATP channels. A trace with the processivity closest to the average for all traces collected for a given condition is highlighted with a thicker line on each panel. The data point and error bars at the top of the panel indicate the mean and variation in the start time of unwinding. (C) Representative traces of wild-type Rep without UV (left, *n* = 26) and with UV (right, *n* = 26). (D) Representative traces of RepΔ2B without UV (left, *n* = 15) and with UV (right, *n* = 15). (E) Unwinding processivity, reported as the average number of base pairs of DNA unwound, with (purple bar) and without UV (gray bar) for RepLA2 (*n*_UV_ = 14, *n* = 12), wild-type Rep (*n*_UV_ = 74, *n* = 118), and RepΔ2B (*n*_UV_ = 20 and *n* = 15). (F) Unwinding speed with and without UV for RepLA2 (*n*_UV_ = 14, *n* = 12), wild-type Rep (*n*_UV_ = 74, *n* = 118), and RepΔ2B (*n*_UV_ = 20 and *n* = 15).

At a constant force, the DNA extension increased over time as the loaded helicase unwound each base pair releasing two nucleotides of ssDNA. Figure 4B shows representative traces of hairpin unwinding by RepLA2 without (left panel) and with (right panel) continuous UV exposure. The traces are aligned such that entry into the ATP channel occurs at *t* = 0. Entry into the ATP channel was determined by recording when the tethered construct crossed the mid-point between the protein and ATP channels. While the locations were constant, and the capillaries used to introduce beads and the tip of the parafilm were used as fiducial markers to initiate the positions, some variability in flow and position is inevitable. Traces in which unwinding begins before or after *t* = 0 most likely represent replicates in which the sample encountered ATP before or after its recorded entry into the ATP channel, which is due to inherent variability from experiment to experiment.

RepLA2 unwinding occurred deeper into the hairpin under the light-on condition compared to no light. For many of the single-molecule trajectories under no-light conditions (Figure 4B, left), a fraction of the hairpin stem was unzipped, followed by a gradual rezipping back to the base. We attribute mid-hairpin reversals to RepLA2 switching to the opposite strand of the hairpin stem and translocating away from the fork junction, allowing the duplex to rezip gradually,^21,45^ behavior which is more consistent with uncrosslinked Rep. In contrast, the hairpin was often unwound completely under light-on conditions (Figure 4B, right). In these cases, gradual rezipping was likely due to helicase translocation past the hairpin loop. Quantification of single-molecule unwinding trajectories shows that under the light-on condition, the average unwinding processivity, or the average number of base pairs unwound per event, was threefold higher compared to the no-light condition (Figure 4E). The unwinding speed was also about 20% higher under the light-on condition compared to no light (Figure 4F).

We performed the same experiments using wild-type Rep (wtRep; Figure 4C) and a variant lacking the 2B subdomain^18^ (RepΔ2B; Figure 4D). wtRep exhibited a similar average processivity as RepLA2 under no-light conditions (Figure 4E). Since the crosslinking efficiency for RepLA2 is estimated to be ∼56% (Figure 2B), we suspect that the RepLA2 unwinding with no UV activation may represent the activity from the fraction of protein that was not crosslinked, which we expect to unwind similarly to wtRep. RepΔ2B is known to exhibit higher processivity than wtRep^18^ and can fully unwind the hairpin, similarly to UV-activated RepLA2 (Figure 4E). We did not observe any significant effect of 365 nm illumination on the average unwinding processivity or speed of either wtRep or RepΔ2B (Figure 4E,F), again confirming that unwinding enhancement did not result from UV-induced heating.

To differentiate the unwinding activity of uncrosslinked RepLA2 that remains in the protein solution, we performed additional optical tweezer experiments with discontinuous UV illumination (Figure S8B and Supporting Information Text). Here, RepLA2 was first illuminated by 465 nm light and then loaded onto the hairpin in the protein channel in the dark (Supporting Information text). The tethered hairpin was next moved into the ATP channel, and UV light was turned on only after a delay of several seconds (ranging between ∼1 and 17 s) (Figures S8B,S9, and Supporting Information Text). To ensure that no RepLA2 was exposed to 365 nm light while in the protein channel, the microfluidic chamber was flushed out (with 40 μL of buffer) after every illumination cycle (Supporting Information text). In addition, all tubes and syringes containing protein were covered to prevent unwanted light exposure. We observed two types of traces: a fraction where unwinding initiated upon exposure to ATP but before UV illumination (Figure S9B, black) and a fraction where unwinding started only after UV exposure (Figure S9B, purple). We attribute the first trace type to the population of RepLA2 that are not crosslinked. Consistent with our interpretation, the average processivity and velocity for this fraction of helicases that can unwind in the absence of light (Figure S9C,D, gray bars) are consistent with the unwinding dynamics of wtRep (Figure 4E,F). In contrast, the fraction of traces that unwound only after exposure to UV, which we believe represent azobenzene crosslinked proteins, exhibit higher average processivity and velocity (Figure S9C,D, purple bars).

## Discussion

Here, we demonstrate several engineered helicases that respond to on-demand modulation with light and have characterized one design, RepLA2, in detail and at the single-molecule level. We demonstrate that the crosslinking of a pair of site-specific cysteine residues with a chemical photoswitch creates a novel type of helicase that can be activated upon UV illumination. While photocontrol has been successfully engineered into the SF1 helicase UvrD,^46^ our conformational control strategy is distinct from their ATP binding gating strategy. Although we do not demonstrate it here, our approach has the novel potential for reversible photocontrol in which different wavelengths of light have the ability to activate or deactivate the helicase. This approach presents an advantage over efforts using a caged lysine in the ATP binding pocket to make a light-activatable helicase where the activation is not reversible and does not have deactivating potential.

Control of the 2B subdomain of Rep helicase using the light-responsive azobenzene moiety will allow more precise study of the relationship between helicase conformation and enzymatic activity. Additionally, spatiotemporal control of a DNA-modifying enzyme may ultimately allow for precise in situ modulation of small DNA structures and, eventually, larger DNA origami-based systems. Helicases have been previously used to control the hybridization state of small DNA constructs to speed up DNA reaction networks^27^ and to process larger in vitro DNA structures.^47^ The growing field of DNA technology may present a future application for modulable helicases. The added functionality by which helicases can be activated or deactivated by an external stimulus may allow for tighter control over the interaction between the helicase and DNA construct. Furthermore, a light-modulated helicase may be useful in nanopore sequencing, where an external light stimulus can be used to change the rate of sequencing or further still, cause a helicase to reverse its motion, and lead to a resequencing of a stretch of DNA for error-correction. In principle, our design strategy may be adopted to create RepLD (LD for light deactivation) where light induces the open conformation that allows strand switching to reverse its movement on DNA.^21^

While we used an azobenzene moiety to synthesize RepLA that is responsive in the UV range, alternate photoswitchable crosslinkers may also be possible. Other azobenzene derivatives have been shown to change both the *cis*-state lifetime^48^ and the wavelength at which the molecule isomerizes, moving away from the UV range of the light spectrum.^48,49^ Further structural modulations of the crosslinker could also expand the distances between which the residue pairs need to fluctuate, if a similar domain motion is to be modified in another family of helicases.

Using single-molecule optical tweezers analysis, we showed that RepLA2 can unwind a hairpin DNA substrate. Interestingly, RepLA2 can bind to the loading site without a free 3′end, although incorporation of azobenzene may create a small topological ring. The topological ring in crosslinked Rep-X prevents it from unwinding without a free 3′-end,^16^ which limits its modularity for some technological applications. We suspect that because the crosslinking sites are much closer to the hinge for the 2B subdomain rotation in RepLAs, single-stranded DNA may gain access to the DNA-binding sites without hindrance from the crosslinker.

Although we observed a strong UV-induced enhancement in unwinding processivity and speed from RepLA2 in our single-molecule experiments, we still observed significant DNA unwinding activities without UV illumination. The processivity, speed, and pattern of unwinding in individual RepLA2 (no UV) traces closely match what we observe for wtRep under monomeric conditions, indicating that this background unwinding may arise from uncrosslinked RepLA2 unwinding as a monomer.

We note that performing bulk unwinding experiments under reducing conditions partially abrogates background unwinding activity, especially for RepLA1 implying that the formation of disulfide bonds may affect a subset of RepLA designs and require further optimization of protein storage and reaction conditions. However, we also note that the effect of disulfide reduction on RepLA2 and RepLA3 is negligible, and single molecule unwinding traces of RepLA2 do not show the same pattern of activity as Rep-X, which we may expect if a disulfide bridge formed between the two cysteine residues. Another possibility to explain background unwinding is that the *trans* state of azobenzene in RepLA2 is not entirely incompatible with the closed conformation of Rep. There may be a conformational heterogeneity where a subpopulation of RepLA2 with *trans* azobenzene exists in the closed conformation, responsible for some of the unwinding activities observed. While we note that there may be some intermolecular crosslinking that occurs during the maleimide reaction, the small percentage of Rep multimers are unlikely to play a large role in unwinding DNA in solution.

## Supporting Information Available

Additional details on the protocol; oligonucleotide sequences for DNA construct synthesis in optical trapping, bulk unwinding experiments, and plasmid mutagenesis; DNA sequences of the handles in the hairpin DNA construct used in single-molecule optical tweezer experiments; chemical structures of bismaleimioethane; uncropped gels showing independent crosslinking reactions; UV–vis spectroscopy; bulk assays; optical setup showing the addition of UV illumination into the custom-built dual-beam optical trap; and schematic representation of the microfluidic chamber during optical trap experiments; and schematic illustration of a protocol for discontinuous in situ UV illumination during a single-molecule optical trapping experiment (PDF)
