# Supplementary material for: Helicase Activity Modulation with On-Demand Light-Based Conformational Control
Source: J Am Chem Soc. 2023 Sep 22;145(39):21253–62. doi: 10.1021/jacs.3c05254 (PMC10557133; doi:10.1021/jacs.3c05254)
Supplement: Supplementary file 1 — ja3c05254_si_001.pdf [file ja3c05254_si_001.pdf]

Supplementary Information for:

Helicase activity modulation with on-demand light-based conformational control

Dmitriy Bobrovnikov[1,‡], Monika A. Makurath[2,3,§,‡], Clara H. Wolfe[1,‡] , Yann R. Chemla[3,4,*], Taekjip Ha[1,5,6,†,*]

[1] Department of Biophysics and Biophysical Chemistry, Johns Hopkins School of Medicine, Baltimore, MD 21205, USA.

[2] Department of Molecular and Integrative Physiology, University of Illinois at Urbana-Champaign, Urbana, Il 61801, USA.

[3] Department of Physics, University of Illinois at Urbana-Champaign, Urbana, Il 61801, USA.

[4]Center for Biophysics and Quantitative Biology, University of Illinois at Urbana-Champaign, Urbana, Il 61801, USA.

[5] Department of Biophysics, Department of Biological Engineering, Johns Hopkins University,

Baltimore, MD 21218, USA

[6] Howard Hughes Medical Institute, Baltimore, MD, 21205, USA

‡These authors contributed equally.

* To whom correspondence should be addressed.
tjha@jhu.edu
ychemla@illinois.edu

This PDF file includes:

Supplementary Information Text
Tables S1-2
Figs. S1 to S9
References for SI reference citations

SUPPLEMENTARY INFORMATION TEXT

DNA construct synthesis

All DNA oligonucleotides for DNA construct synthesis are listed in Table S1. The DNA constructs for optical trapping experiments were synthesized as previously described[1]. The final DNA hairpin construct was stored at 100-200 ng/µL, in 2.5 µM aliquots at -30°C. The construct contains an internal 1′,2′-Dideoxyribose Spacer (idSp) to prevent the possibility of unwinding the DNA handle past the hairpin. Short reporter oligonucleotides for bulk assays using both FRET and the fluorescein/quencher system were assembled as follows: complementary strands were mixed in equimolar amounts, then annealed by heating the constructs to 98°C and cooling slowly to 25°C over the course of 45 minutes.

Residue selection based on solvent accessibility criteria

We examined the distribution of native cysteines in the crystal structure of a Rep molecule (Figure S1A). The cysteines are at distinct spatial locations, and substitutes of these residues to create a cysteine-free Rep are unlikely to affect the structure or the function of the protein. Thus, we used a pET28a vector containing a 6X His tagged, cysteine-free Rep construct as described previously[2, 3] (C18L, C43S, C167V, C178A, C612A) and mutated select candidate residues (Fig. 1D, Fig. S1D) to cysteines to enable crosslinking with maleimide-functionalized chemical crosslinkers (Fig. S1B). Mutagenic primers are listed in Table S1.

Candidate residue pairs were chosen by appropriate distance criteria such that the inter-residue distance is between 16 and 18 Å in the open conformation and between 5 and 12 Å in the closed conformation. Residue pairs that did not meet these criteria were thrown out. Residue pairs that fit the distance criteria were further filtered by their solvent accessibility[4, 5] (Fig. S1C-D). We calculated the accessible surface area of each residue in its "cysteine replaced" state and prioritized residue pairs with the greatest solvent accessibility for the least accessible member of the pair. Residue pairs were manually examined to ensure that they would not disrupt DNA or ATP binding[3, 6, 7].

Crosslinking quantification

Crosslinked helicases exhibit characteristic band retardation when run using reducing SDS-PAGE. Figure S2 shows gels for three independent crosslinking reactions. Gels 1 and 2 were run on 4-20% gradient gels (Mini-PROTEAN® TGX™, BioRad) for 10 minutes at 100 V followed by 55 minutes at 160 V. Gels were washed and stained for 1 hour at room temperature with SimplyBlue™ Safe Stain (Invitrogen) then destained at 4°C overnight and for 1 hour at room temperature prior to imaging. Gel 3 was run on a gradient gel (Any kD™ Mini-PROTEAN® TGX™, BioRad) for 1 hour at 150 V then washed and stained with GelCode™ Blue Safe Protein stain at room temperature for 30 minutes, then destained overnight at 4°C and for 1 hour at room temperature prior to imaging. Band areas were quantified using ImageJ[8].

Characterization of RepLA by UV-Vis spectroscopy

Samples were subject to UV-Vis spectroscopy as described in the main text either at equilibrium (Fig. S3A) or after varying exposures to 455-nm light (Fig. S3B). Freshly thawed helicase samples were treated in thin-walled PCR tubes with an initial irradiation with 455-nm light for 1 min to ensure that protein-incorporated azobenzene was consistently in the *trans* form and an initial measurement was taken (Fig. S4). Samples were then irradiated for 30 seconds with 365-nm light in thin-walled PCR tubes to convert azobenzene to the *cis* form and allowed to isomerize to the *trans* form at room temperature, in the absence of light. A corresponding sample of RepLA2 was allowed to isomerize to the *trans* form in the presence of ambient light. Time points were measured in triplicate and the line plots of absorbance vs. wavelength depict the average absorbance for each replicate.

RepLA2 characterization with ensemble measurements and FRET

To confirm that the helicase fully unwinds a short fluorescent DNA reporter and to provide temporal data for the kinetics of unwinding, we repeated bulk unwinding assays using a short DNA construct in which one strand is labelled at the 5′-end with a Cy5 fluorophore and contains a 10 nucleotide 3′-overhang to load the helicase. The other strand is labelled at the 3′-end with a Cy3 fluorophore (Table S1, Fig. S6A). When the strands are annealed, they exhibit high FRET due to the proximity of the fluorophores. When the strands are denatured, the FRET signal decreases. We tested RepLA2 helicases that were not crosslinked, were crosslinked with azobenzene, or were crosslinked with ethane. All helicases were initially treated with 1 minute of 455-nm light to ensure that azobenzene is in its *trans* form. A subset of azobenzene crosslinked proteins was additionally treated with 30 seconds of UV light to isomerize the crosslinker to the *cis* form and activate the protein. Helicases were added to 200 μL of

reaction solution in a glass cuvette for a final concentration of 20 nM. Reaction solution consisted of 10 mM $MgCl_2$, 100 mM TrisHCl (pH 8.0), 10% glycerol, 100 mM DTT, and 15 mM NaCl. Reactions were initiated by the simultaneous addition of the DNA reporter construct and ATP for final concentrations of 50 nM and 1 mM respectively. Fluorescent emission at 570-nm and 667-nm was recorded every 2.5 seconds after 550-nm excitation. Signals for individual traces were denoised using a rolling window of 25 seconds.

UV activation setup in the single-molecule optical trap measurements

To quantify the effect of UV on the activity of the RepLA2 variant, we designed an optical setup that would ensure a consistent UV illumination between experiments. We illuminate the sample plane by epi-illumination to redistribute the light evenly throughout the working area of the sample chamber. We inserted the 365-nm UV light into the beam path by means of a beam-splitting cube (Fig. S7A, Fig. S7B). We collimated the light beam (Fig. S7A, Fig. S7C) and used a lens with a matching working distance to focus the light at the back-focal plane of the front objective (Fig. S7A, FO). We measured the UV power after the light exited the front object (FO), which corresponds to the location of the sample plane. A power adapter allowed us to control the UV power at the sample plane. We decided to work with minimal power (1-10 mW) to prevent the unwanted effects of UV light such as heating or photodamage. Figure S7D shows a picture of the 365-nm beam of light at the specimen plane. We fine-tuned the alignment, ensuring a uniform distribution of light at the specimen plane, by imaging the light onto a CCD camera (CCDC, Fig. S7A).

RepLA activation workflow in a single-molecule microfluidic chamber

The microfluidic chamber for single-molecule optical trap measurements consisted of four buffer channels, two outer channel and two inner channels (Fig. S8, bottom right, outer – yellow and green, inner – blue and red). As described in Materials and Methods, the inner channels contained protein (top, blue) and ATP (bottom, red) and the outer channels contained streptavidin (top) and anti-digoxigenin (bottom) beads. The outer channels are connected to the inner channels via thin (100-μm OD) glass capillaries. At the beginning of each experiment, we trap a streptavidin bead coated with DNA first, followed by an anti-digoxigenin bead. Additionally, the traps are calibrated with the captured beads, and moved to a desired location within the chamber. After calibration, we form a DNA tether and ensure that the mechanical response of the DNA molecule matches the predicted response. Throughout this process, we visually inspect the trapped beads, continuously illuminating the sample

plane with 430 nm for up to 15 min. We then turn of the light 465-nm light (all other light apart from the 1064-nm trap light are completely blacked out) and proceed to turn on the UV (365 nm) in an either continuous or discontinuous mode (described below).

RepLA2 unwinding using continuous illumination in single-molecule measurements

The RepLA2 protein channel was illuminated with 465-nm light as described above. The 465-nm light was then turned off, and the 365-nm light was turned on for the remainder of the experiment with a given bead pair (5-30 min), which involved repetitive cycling of loading the protein by placing the DNA-tethered beads for 5 s in the protein channel and then moving for 10-30 s to the ATP channel.

RepLA2 unwinding using discontinuous illumination in single-molecule measurements

The RepLA2 protein was illuminated with 465-nm light as described above. After trap calibration and verification of the DNA tether, we turned off the 465-nm light, waited 5 seconds, and moved to the ATP channel. For one population of RepLA2, we saw immediate unwinding in dark (Fig. S9B, black), which--based on the results from SDS electrophoresis, bulk assays, and continuous experiments—we attributed to uncrosslinked population of RepLA2. If no unwinding was observed upon entering ATP, we turned on the UV after a time delay, for 5-60 s. For a small fraction of attempts (~1:80), we observed unwinding within seconds of turning on the UV (Fig. S9B, right), which we attribute to the crosslinked RepLA2 population. After each attempt at discontinuous UV illumination, we flushed 40 μL of the solutions in the middle channels (protein and ATP) to remove any UV-activated protein. We then proceed with the experiments as described in sections above, which start with 465-nm illumination.

We performed the discontinuous experiment 5 times to acquire the dataset. The first four times, we manually tracked the timing of UV light with a timer. For the last experiment, we tracked the 365-nm signal using the data collection software with 0.01 s precision. We used the three traces acquired with precise UV tracking to calculate the activation time $T = t_{\mathrm{UV}} - t_{\mathrm{ATP}}$, which allowed us to determine that it takes 3.2 ± 0.4 s to activate RepLA2 under our experimental conditions. We aligned the traces in Figure S9B (purple) such that t = 0 corresponds to the time when UV is turned on, which emphasizes that unwinding occurs only after turning on UV for a population of RepLA2 and shows how long it takes to activate the protein.

SUPPLEMENTARY TABLE

Name	Assay	Sequence
LH forward primer	Optical trapping hairpin	5′-/5Biosg/TGAAGTGGTGGCCTAACTACGCAA-3′
LH reverse primer	Optical trapping hairpin	5′-CAAGCCTATGCCTACAGCAT-3′
RH forward primer	Optical trapping hairpin	5′-/5DigN/CAACAACGTTGCGCAAACT-3′
RH reverse primer	Optical trapping hairpin	5′/5Phos/TTGAAATACCGACCGCTCAGCTATCAGCCTTTTTTTTTT/ idSp/CTCTGACACATGCAGCTCCC-3′
Hairpin insert	Optical trapping hairpin	5′/5Phos/CCTGGGGCTGATAGCTGAGCGGTCGGTATTTCAAAAGT CAACGTACTGATCACGCTGGATCCTAGAGTCAACGTACTGATC ACGCTGGATCCTATTTTTAGGATCCAGCGTGATCAGTACGTTGA CTCTAGGATCCAGCGTGATCAGTACGTTGACTT-3′
Fluorescein/ Quencher	Fluorescein bulk unwinding	5′/5Fluor/TGCCTCGCTGCCGTCGCCA/3Dab/3′
dT10 28mer	Fluorescein bulk unwinding	5′TGGCGACGGCAGCGAGGCTTTTTTTTTT3′
Cold Strand	Fluorescein bulk unwinding	5′TGCCTCGCTGCCGTCGCCA3′
FRET dT10 28mer	FRET bulk unwinding	5′/5Cy5/GCCTCGCTGCCGTCGCCATTTTTTTTTT3′
FRET 18mer	FRET bulk unwinding	5′TGGCGACGGCAGCGAGGC/3Cy3Sp/3′
D109C Fwd	Site-directed mutagenesis	5′CAGCCTGTTTTGCGATACCGATC3′
D109C Rvs	Site-directed mutagenesis	5′AAGTTCGCTTTCATACCC3′
T111C Fwd	Site-directed mutagenesis	5′GTTTGACGATTGCGATCAGCTGG3′
T111C Rvs	Site-directed mutagenesis	5′AGGCTGAAGTTCGCTTTC3′
T375C Fwd	Site-directed mutagenesis	5′CAGCGGTGGCTGCAGCTTCTTTAG3′
T375 Rvs	Site-directed mutagenesis	5′ATTTTGTACGGAATACGGTTTTG3′
S379C Fwd	Site-directed mutagenesis	5′CAGCTTCTTTTGCCGTCCGGA3′
S379C Rvs	Site-directed mutagenesis	5′GTGCCACCGCTGATTTG3′
R380C Fwd	Site-directed mutagenesis	5′CTTCTTTAGCTGTCCGGAAATTAAAGATC3′
R380C Rvs	Site-directed mutagenesis	5′CTGGTGCCACCGCTGATT3′

Table S1. Oligonucleotide sequences for DNA construct synthesis in optical trapping, bulk unwinding, experiments, and plasmid mutagenesis. All sequences are in an IDT format. Key: LH: Left Handle, RH: Right Handle, 5Biosg: 5′ Biotin, 5Phos: 5′ Phosphorylation, 5DigN: 5′ Digoxigenin, idSp: internal 1′,2′-Dideoxyribose, 5Fluor: 5′ Fluorescein, 3Dab: 3′ Dabcyl, 5Cy5: 5′ Cy5, 3Cy3Sp: 3′ Cy3.

Handle	Sequence
Right	CTCTGACACATGCAGCTCCCGGAGACGGTCACAGCTTGTCTGTAAGCGGATGCCGGGAGCAGAC AAGCCCGTCAGGGCGCGTCAGCGGGTGTTGGCGGGTGTCGGGGCGCAGCCATGACCCAGTCAC GTAGCGATAGCGGAGTGTATACTGGCTTAACTATGCGGCATCAGAGCAGATTGTACTGAGAGTG CACCATATGCGGTGTGAAATACCGCACAGATGCGTAAGGAGAAAATACCGCATCAGGCGCTCT TCCGCTTCCTCGCTCACTGACTCGCTGCGCTCGGTCGTTCGGCTGCGGCGAGCGGTATCAGCTCA CTCAAAGGCGGTAATACGGTTATCCACAGAATCAGGGGATAACGCAGGAAAGAACATGTGAGC AAAAGGCCAGCAAAAGGCCAGGAACCGTAAAAAGGCCGCGTTGCTGGCGTTTTTCCATAGGCT CCGCCCCCCTGACGAGCATCACAAAAATCGACGCTCAAGTCAGAGGTGGCGAAACCCGACAGG ACTATAAAGATACCAGGCGTTTCCCCCTGGAAGCTCCCTCGTGCGCTCTCCTGTTCCGACCCTGC CGCTTACCGGATACCTGTCCGCCTTTCTCCCTTCGGGAAGCGTGGCGCTTTCTCATAGCTCACGCT GTAGGTATCTCAGTTCGGTGTAGGTCGTTCGCTCCAAGCTGGGCTGTGTGCACGAACCCCCCGTT CAGCCCGACCGCTGCGCCTTATCCGGTAACTATCGTCTTGAGTCCAACCCGGTAAGACACGACT TATCGCCACTGGCAGCAGCCACTGGTAACAGGATTAGCAGAGCGAGGTATGTAGGCGGTGCTA CAGAGTTCTTGAAGTGGTGGCCTAACTACGGCTACACTAGAAGGACAGTATTTGGTATCTGCGC TCTGCTGAAGCCAGTTACCTTCGGAAAAAGAGTTGGTAGCTCTTGATCCGGCAAACAAACCACC GCTGGTAGCGGTGGTTTTTTTGTTTGCAAGCAGCAGATTACGCGCAGAAAAAAAGGATCTCAAG AAGATCCTTTGATCTTTTCTACGGGGTCTGACGCTCAGTGGAACGAAAACTCACGTTAAGGGATT TTGGTCATGAGATTATCAAAAAGGATCTTCACCTAGATCCTTTTAAATTAAAAATGAAGTTTTAA ATCAATCTAAAGTATATATGAGTAAACTTGGTCTGACAGTTACCAATGCTTAATCAGTGAGGCA CCTATCTCAGCGATCTGTCTATTTCGTTCATCCATAGTTGCCTGACTCCCCGTCGTGTAGATAACT ACGATACGGGAGGGCTTACCATCTGGCCCCAGTGCTGCAATGATACCGCGAGACCCACGCTCAC CGGCTCCAGATTTATCAGCAATAAACCAGCCAGCCGGAAGGGCCGAGCGCAGAAGTGGTCCTG CAACTTTATCCGCCTCCATCCAGTCTATTAATTGTTGCCGGGAAGCTAGAGTAAGTAGTTCGCCA GTTAATAGTTTGCGCAACGTTGTTG
Left	TGAAGTGGTGGCCTAACTACGGCTACACTAGAAGGACAGTATTTGGTATCTGCGCTCTGCTGAA GCCAGTTACCTTCGGAAAAAGAGTTGGTAGCTCTTGATCCGGCAAACAAACCACCGCTGGTAGC GGTGGTTTTTTTGTTTGCAAGCAGCAGATTACGCGCAGAAAAAAAGGATCTCAAGAAGATCCTT TGATCTTTTCTACGGGGTCTGACGCTCAGTGGAACGAAAACTCACGTTAAGGGATTTTGGTCATG AGATTATCAAAAAGGATCTTCACCTAGATCCTTTTAAATTAAAAATGAAGTTTTAAATCAATCTA AAGTATATATGAGTAAACTTGGTCTGACAGTTACCAATGCTTAATCAGTGAGGCACCTATCTCA GCGATCTGTCTATTTCGTTCATCCATAGTTGCCTGACTCCCCGTCGTGTAGATAACTACGATACG GGAGGGCTTACCATCTGGCCCCAGTGCTGCAATGATACCGCGAGACCCACGCTCACCGGCTCCA GATTTATCAGCAATAAACCAGCCAGCCGGAAGGGCCGAGCGCAGAAGTGGTCCTGCAACTTTA TCCGCCTCCATCCAGTCTATTAATTGTTGCCGGGAAGCTAGAGTAAGTAGTTCGCCAGTTAATAG TTTGCGCAACGTTGTTGCCATTGCTGCAGGCATCGTGGTGTCACGCTCGTCGTTTGGTATGGCTTC ATTCAGCTCCGGTTCCCAACGATCAAGGCGAGTTACATGATCCCCCATGTTGTGCAAAAAAGCG GTTAGCTCCTTCGGTCCTCCGATCGTTGTCAGAAGTAAGTTGGCCGCAGTGTTATCACTCATGGTT ATGGCAGCACTGCATAATTCTCTTACTGTCATGCCATCCGTAAGATGCTTTTCTGTGACTGGTGA GTACTCAACCAAGTCATTCTGAGAATAGTGTATGCGGCGACCGAGTTGCTCTTGCCCGGCGTCA ACACGGGATAATACCGCGCCACATAGCAGAACTTTAAAAGTGCTCATCATTGGAAAACGTTCTT CGGGGCGAAAACTCTCAAGGATCTTACCGCTGTTGAGATCCAGTTCGATGTAACCCACTCGTGC ACCCAACTGATCTTCAGCATCTTTTACTTTCACCAGCGTTTCTGGGTGAGCAAAAACAGGAAGG CAAAATGCCGCAAAAAAGGGAATAAGGGCGACACGGAAATGTTGAATACTCATACTCTTCCTT TTTCAATATTATTGAAGCATTTATCAGGGTTATTGTCTCATGAGCGGATACATATTTGAATGTATT TAGAAAAATAAACAAATAGGGGTTCCGCGCACATTTCCCCGAAAAGTGCCACCTGACGTCTAA GAAACCATTATTATCATGACATTAACCTATAAAAATAGGCGTATCACGAGGCCCTTTCGTCTTCA AGAATTCTCATGTTTGACAGCTTATCATCGATAAGCTTTAATGCGGTAGTTTATCACAGTTAAATT GCTAACGCAGTCAGGCACCGTGTATGAAATCTAACAATGCGCTCATCGTCATCCTCGGCACCGT CACCCTGG

Table S2. DNA sequences of the handles in the hairpin DNA construct used in single-molecule optical tweezer experiments.

SUPPLEMENTARY FIGURES

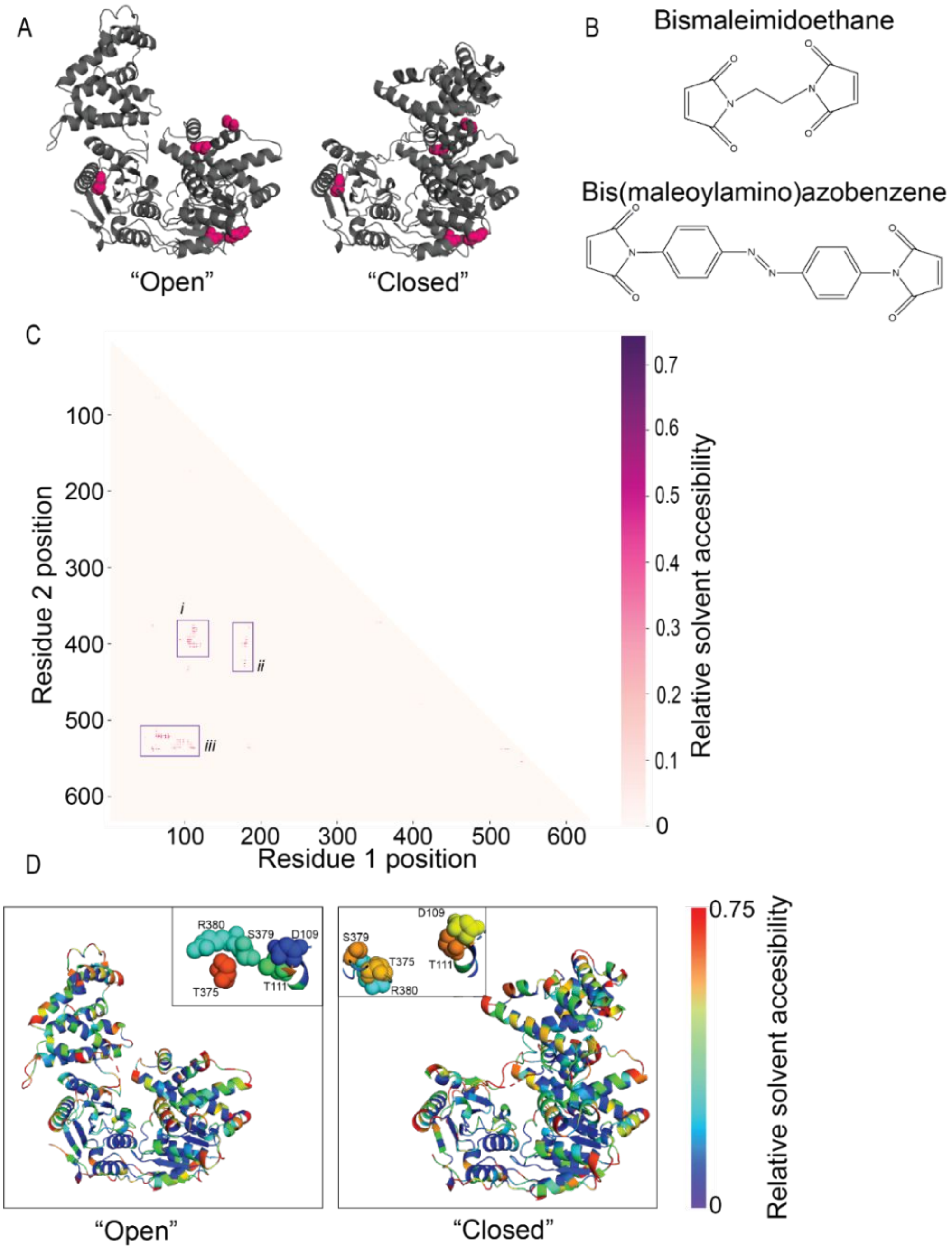

Figure S1. (A) Rep in the open and closed conformations with native cysteines highlighted in magenta. (B) Chemical structures of bismaleimioethane (henceforth referred to as ethane) and the *trans* isomer of bis(maleoylamino)azobenzene (henceforth referred to as azobenzene). (C) Residue pairs were selected such that they match the distance criteria and optimize solvent accessibility to aid in crosslinking. Residues pairs that match the distance parameters generally clustered in three regions (depicted here as *i, ii, iii*). These residues are further filtered to maximize the solvent accessibility of the least accessible member of the pair. (D) Crystal structures of the 'open' and 'closed' forms of Rep colored by their relative solvent accessibility. Insets show the residues that were selected for RepLA1, RepLA2, and RepLA3.

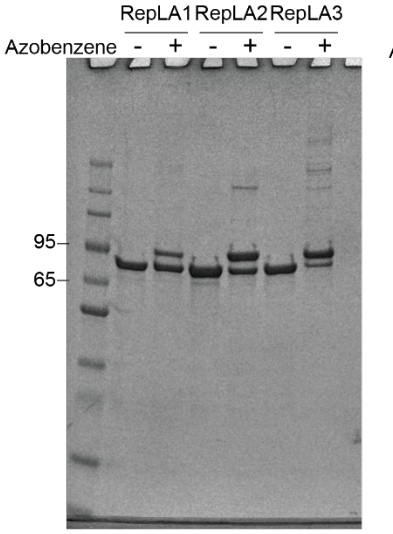

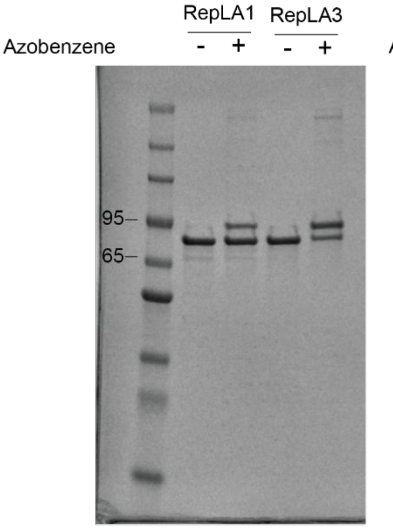

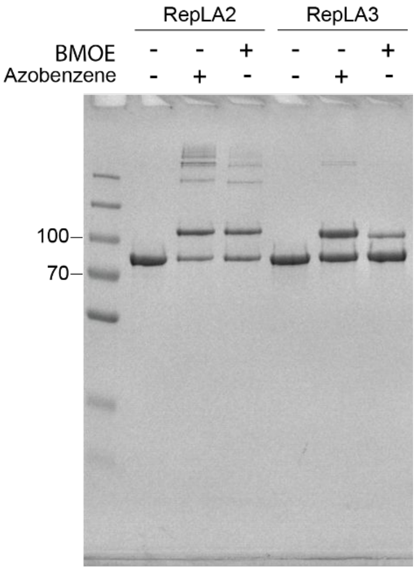

Azobenzene band %			
Band	LA1	LA2	LA3
Non-specific	0.60	5.82	15.82
Crosslinked	35.13	56.53	61.61
Un-crosslinked	64.26	37.64	22.55

Azobenzene band %		
Band	LA1	LA3
Non-specific	8.60	3.39
Crosslinked	31.92	60.58
Un-crosslinked	67.25	36.01

Azobenzene band %		
Band	LA2	LA3
Non-specific	35.13	2.16
Crosslinked	42.28	47.00
Un-crosslinked	22.58	50.82

Ethane band %		
Band	LA2	LA3
Non-specific	8.74	0.0
Crosslinked	49.10	19.83
Un-crosslinked	42.15	80.17

Figure S2. Uncropped gels showing independent crosslinking reactions. The quantification of gel bands is shown as percentage of total band area for each lane. Crosslinked proteins exhibit characteristic band retardation. Crosslinking efficiency as calculated by the percentage of total band area in the upper band is consistently between 30% and 60%.

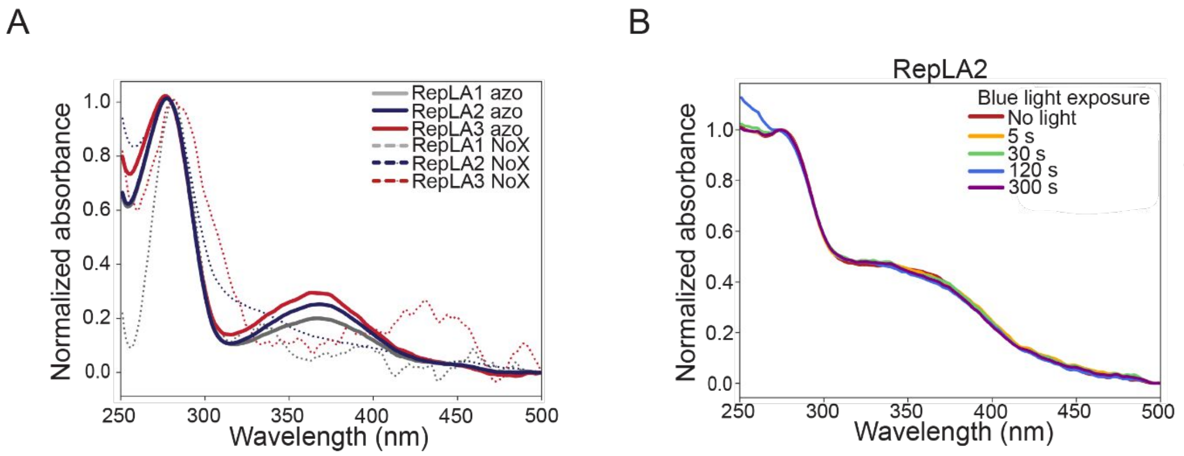

Figure S3. (A) UV-Vis spectroscopy comparing RepLA designs crosslinked with azobenzene to un-crosslinked helicases. The un-crosslinked proteins do not show the characteristic absorbance peak at 365-nm. (B) UV-vis spectroscopy with varying time exposures of a fresh protein sample to 455-nm light. Exposure of protein that has had no previous UV exposure to blue light does not change the absorbance peak at 365-nm. We therefore assume that the azobenzene is predominantly in the *trans* form throughout the crosslinking reaction and remains in this state in all unmodified reactions.

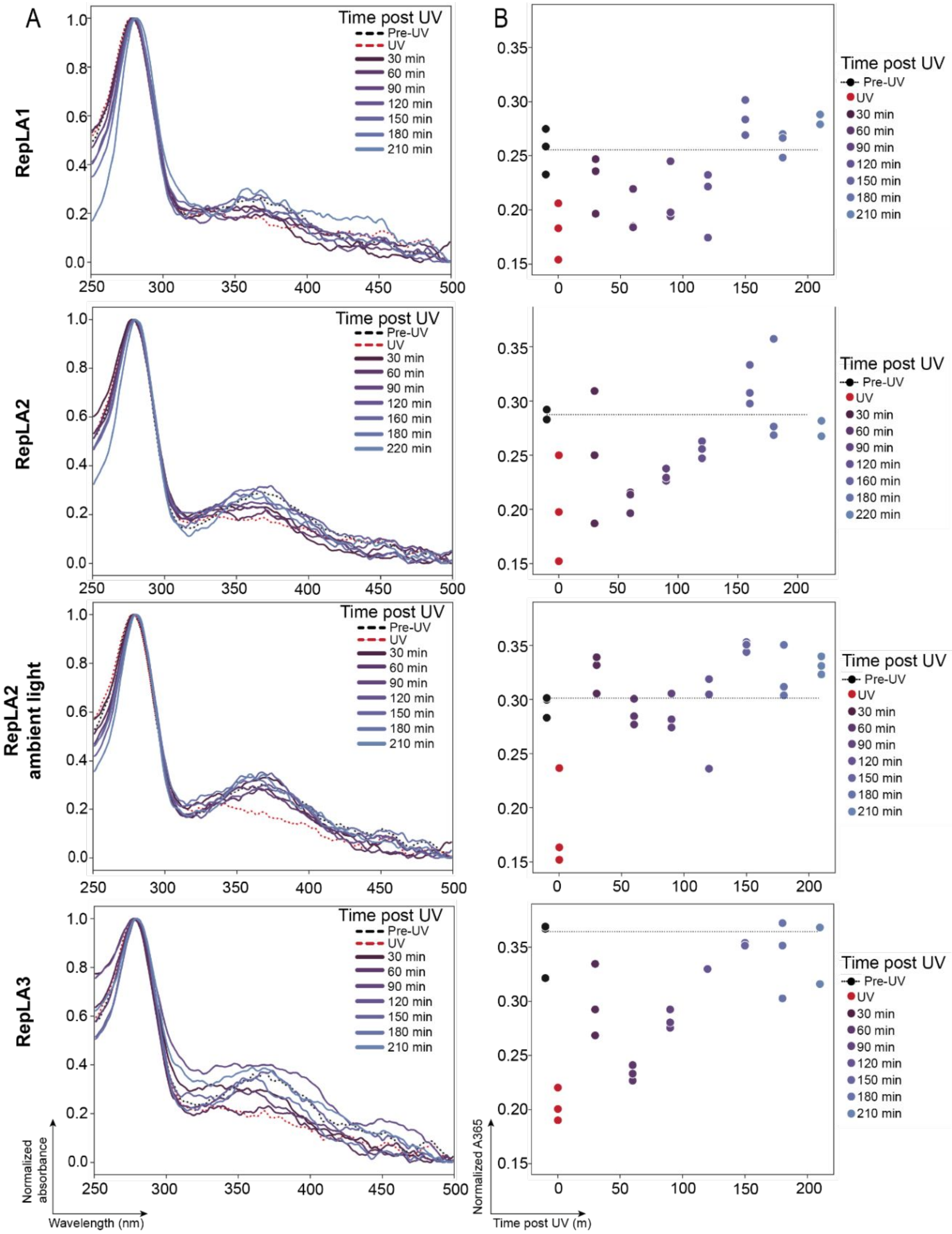

Figure S4. (A) UV-Vis spectroscopy of RepLA1, RepLA2, and RepLA3 post irradiation with 30 seconds of UV light (red dotted line). All samples show characteristic reversion to the *trans* form (black dotted line) over time. Traces depict the mean of the replicates shown in (B). (B) The kinetics of reversion for each sample is displayed as the normalized absorbance at 365-nm over the time course. The dashed black line depicts the average absorbance at 365-nm for the samples not exposed to UV light.

Approximate half-lives for isomerization to the *trans* form are: 75 minutes for RepLA1, 82 minutes for RepLA2, 34 minutes for RepLA2 at ambient light, 61 minutes for RepLA3.

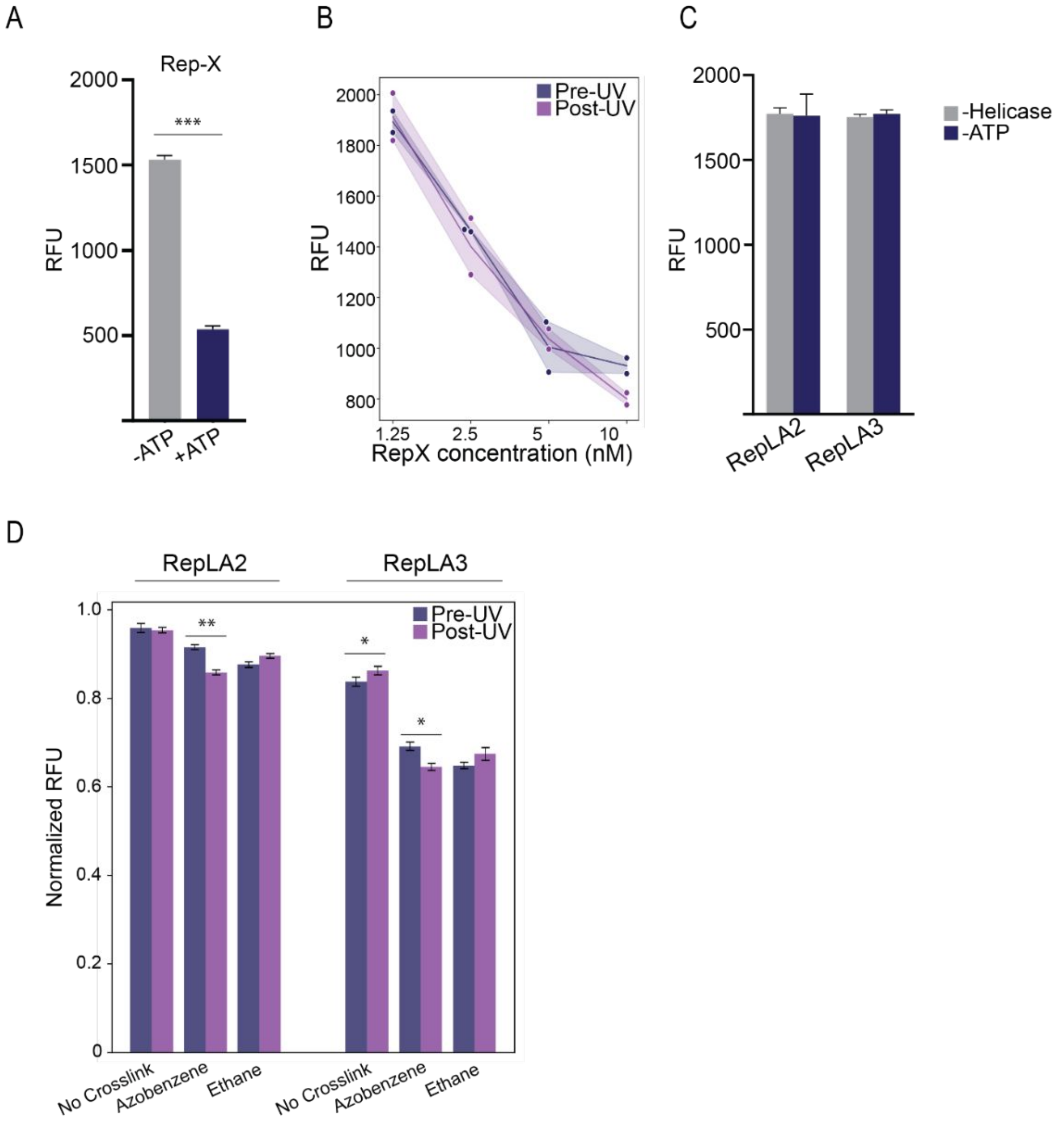

Figure S5. (A) Bulk assay depicted in Fig. 3 using Rep-X protein in the presence (blue bar, *n* = 8) and absence (grey bar, *n* = 8) of ATP. (B) Bulk assay as depicted in Fig. 3 showing increased DNA unwinding with increasing concentrations of Rep-X protein. Individual experimental replicates (*n* = 2) depicted as circles with the midpoint and range depicted as a solid line and shaded bar. (C) Baseline values of the bulk activity assay showing no difference in relative fluorescent units for samples containing full reaction buffer without helicase (grey bar, *n* = 3) and samples containing the protein and reaction buffer without ATP (blue bar, *n* = 3 for each). Data displayed as mean and standard error. $p > 0.05$ for all datasets as determined by two-sided independent t-test. (D) Bulk assay unwinding assay for RepLA2 and RepLA3 completed under reducing conditions as previously described for RepLA1 (Fig. 3). Data displayed as mean and standard error. Paired t-tests were used to calculate the significance of the differences between untreated (*n* = 4, blue bars) and UV treated samples (*n* = 4, purple bars) for each condition ** $p < 0.01$, * $p < 0.05$, p-values > 0.05 are not marked.

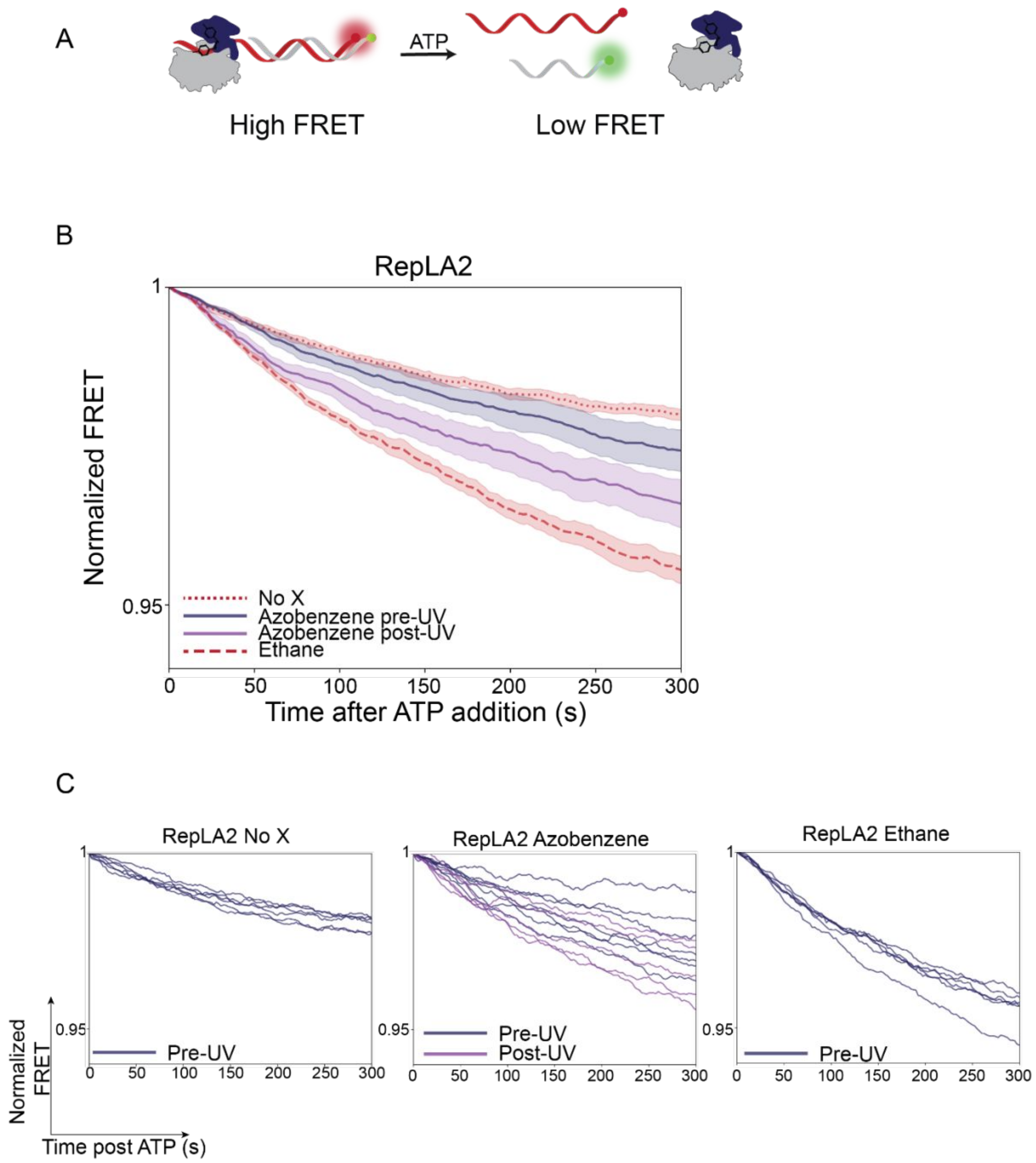

Figure S6. (A) Schematic representation of bulk FRET experiments. Decrease in the FRET signal corresponds to successful unwinding of the DNA by RepLA2. (B) FRET traces show decrease in FRET over time after the addition of the DNA reporter and ATP to the helicase solution, corresponding to unwinding of the DNA construct by uncrosslinked RepLA2, or RepLA2 crosslinked with azobenzene or ethane. Traces are represented as the average and standard error of several replicates (n = 6 for uncrosslinked RepLA2, n = 7 for azobenzene crosslinked RepLA2 without UV, n = 5 for azobenzene crosslinked RepLA2 + UV, n = 6 for RepLA2 crosslinked with ethane. (C) Individual FRET traces for un-crosslinked RepLA2, azobenzene-crosslinked RepLA2 with and without UV exposure, and RepLA2 crosslinked with ethane. All traces were normalized to the FRET signal recorded immediately after the addition of ATP and DNA reporter.

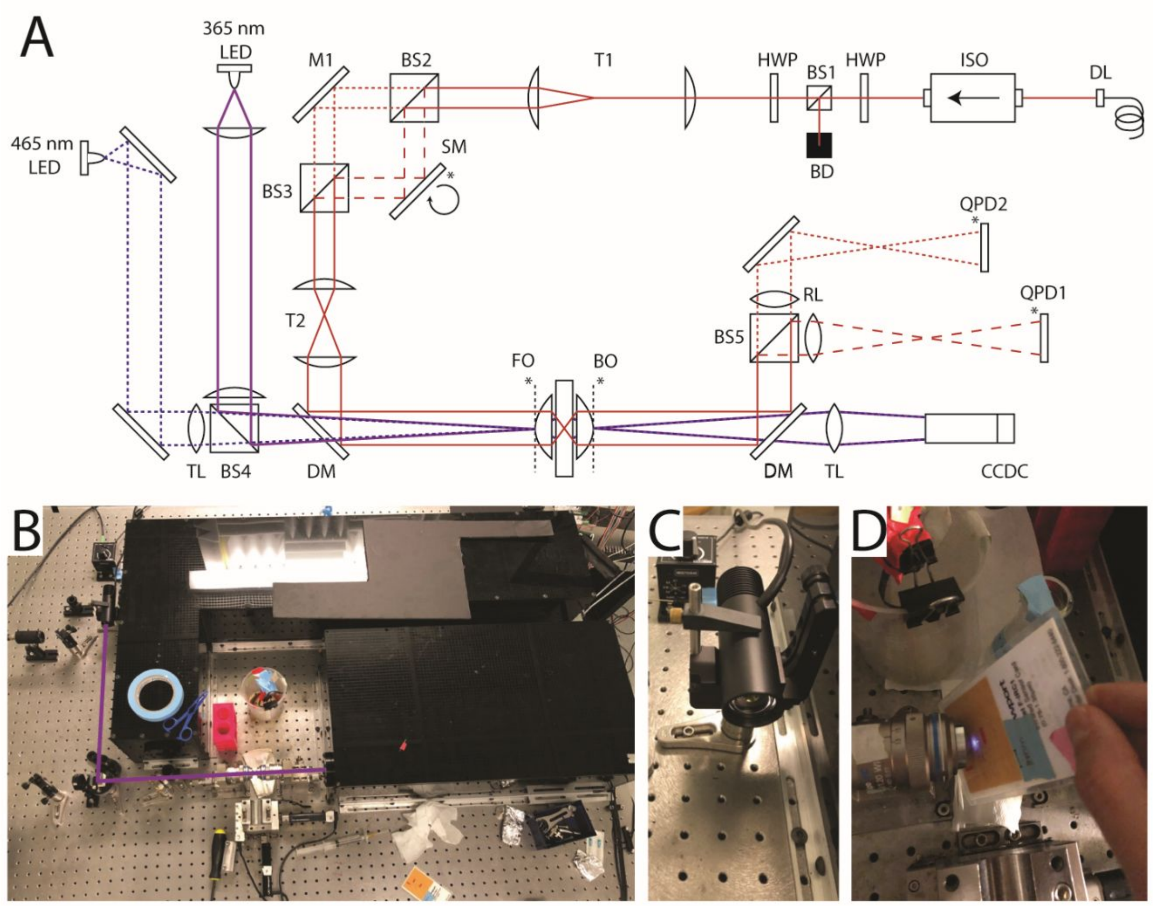

Figure S7. (A) Optical setup showing the addition of UV illumination into the custom built dual-beam optical trap (image adapted from Suksombat, S., 2015[9]). The UV illumination setup incorporates a 365-nm LED (M354 LP1C1 ThorLabs) in the optical path. The LED and the power adapter were placed near the bright-field 465-nm visible LED source (left). A set of two lenses collimate the light and focus it onto the back-focal plane of the front objective to epi-illuminate the specimen plane. The UV light was inserted into the optical path by a beam-splitting cube (BS4). Key: DL – 1064-nm Laser source, ISO - Optical Isolator, HWP - Half-wave Plate, BS - Polarized Beam Splitter, BD - Beam Dump, T - Telescope, M - Mirror, SM - Steering Mirror, SH - Shutter, DM - Dichroic Mirror, FO - Front Objective, BO - Back Objective, TL - Tube Lens, RL - Relay Lens, CCDC - Camera, QPD - Quadrant Photodiode Detector. (B) Picture of the optical table with a purple line drawn to highlight the path of 365-nm UV illumination. (C) Picture of the 365-nm LED. (D) 365-nm illumination after the front objective.

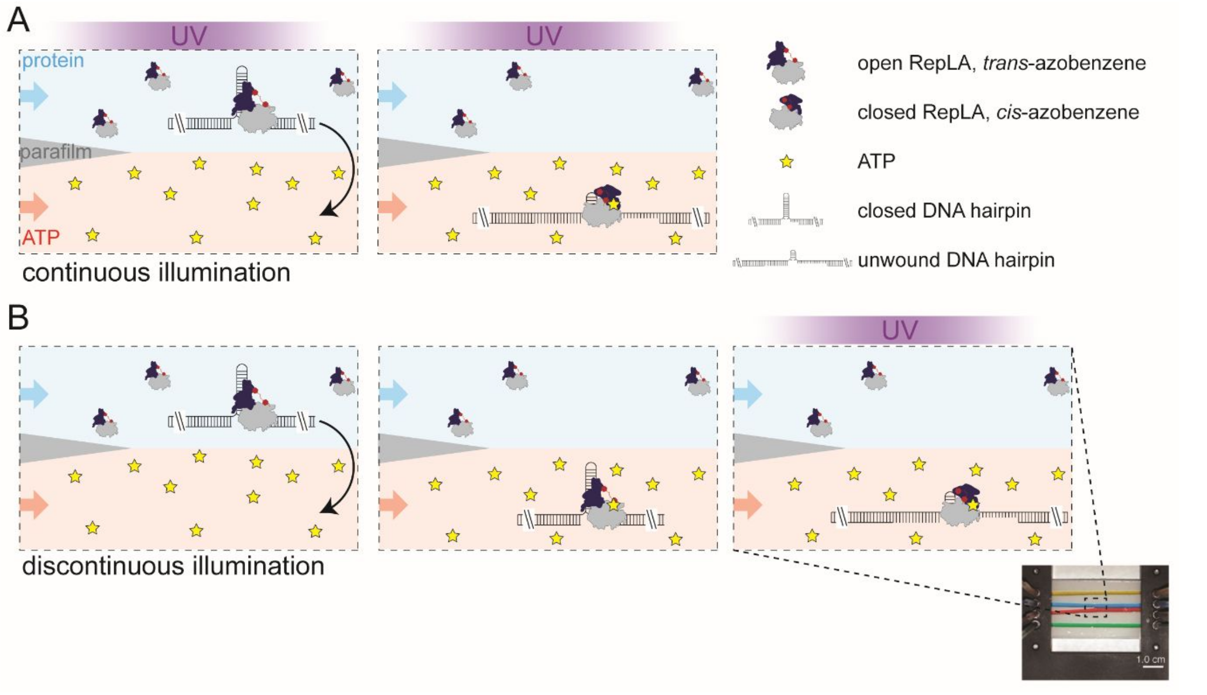

Figure S8. Schematic representation of the microfluidic chamber during optical trap experiments. (A) The protein is loaded in the protein channel (5 s) and moved to ATP channel (10-30 s) under continuous UV illumination. (B) In discontinuous illumination experiments, the protein is loaded in the protein channel (5 s) and moved to the ATP channel (10-60 s) without any prior exposure to 365-nm light. The UV is turned on seconds after moving to the ATP channel. Picture of the actual microfluidic chamber is added for the reference (bottom right).

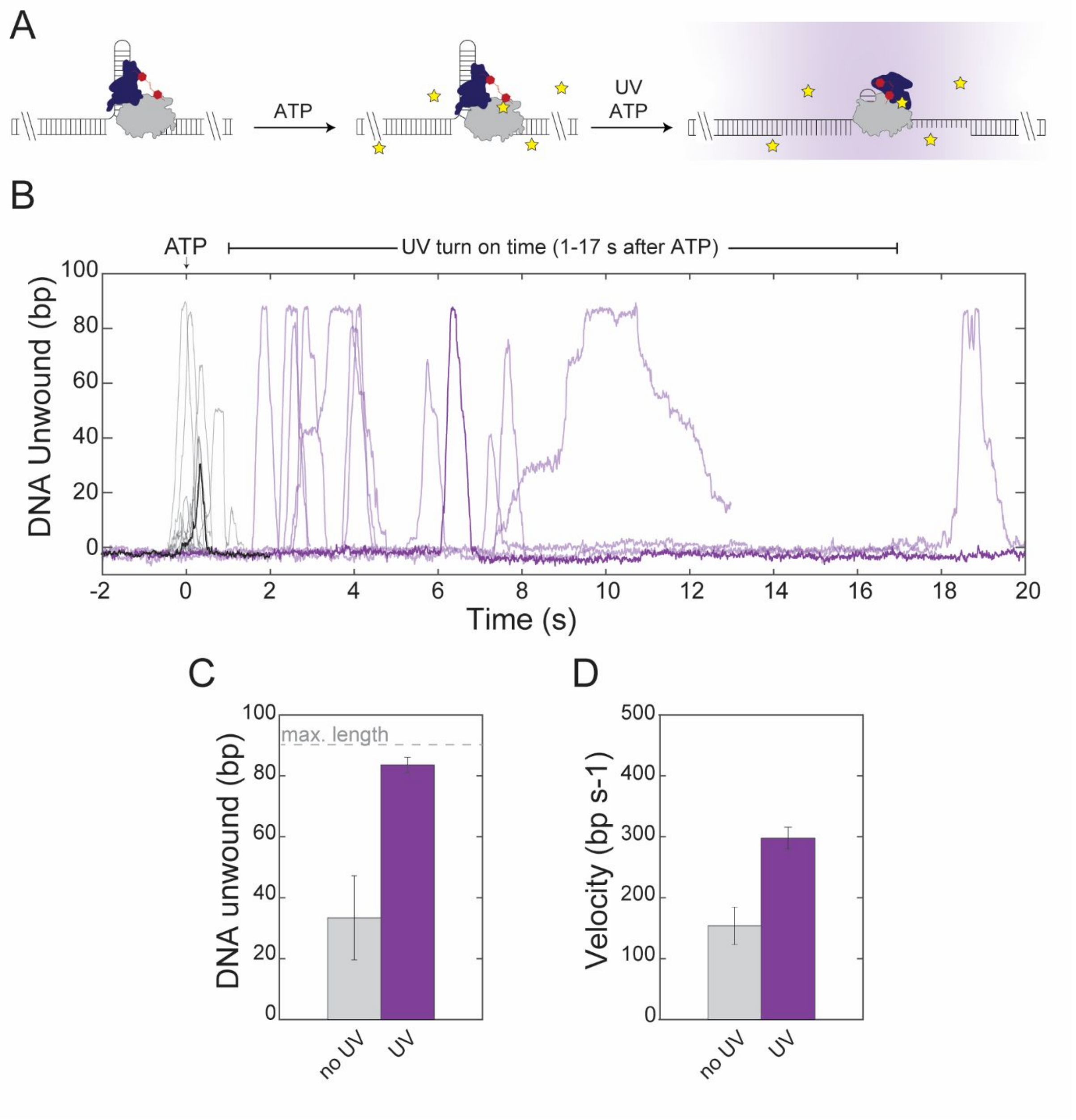

Figure S9. (A) Schemic illustration of a protocol for discontinuous *in situ* UV illumination during a single-molecule optical trapping experiment (details described in materials and methods and SI text). RepLA2 is loaded in the protein channel, then moved to the ATP channel, and finally illuminated with 1-10 mW of UV light. (B) Representative traces that initiated unwinding upon addition of ATP but before any exposure to UV (black). Unwinding traces that initiated only after UV exposure (purple); no unwinding occurred upon entering ATP in the absence of UV, and unwinding proceeded on average 3.2 ± 0.4 s after UV illumination (see SI text). Here, t = 0 marks the time when the protein enters the midpoint between the protein and ATP channels. (C) Unwinding processivity, reported as the average number of base pairs of DNA unwound, for unwinding that initiated only after UV exposure (n_{UV} = 13, purple bar) and for unwinding that initiated prior to UV exposure (n = 15, gray bar). (D) Unwinding speed for the same data sets (n_{UV} = 13, purple bar; and n = 15, gray bar).

References

(1) Makurath, M. A.; Whitley, K. D.; Nguyen, B.; Lohman, T. M.; Chemla, Y. R. Regulation of Rep helicase unwinding by an auto-inhibitory subdomain. *Nucleic Acids Res* **2019**, *47* (5), 2523-2532. DOI: 10.1093/nar/gkz023.
(2) Arslan, S.; Khafizov, R.; Thomas, C. D.; Chemla, Y. R.; Ha, T. Protein structure. Engineering of a superhelicase through conformational control. *Science* **2015**, *348* (6232), 344-347. DOI: 10.1126/science.aaa0445.
(3) Rasnik, I.; Myong, S.; Cheng, W.; Lohman, T. M.; Ha, T. DNA-binding orientation and domain conformation of the E. coli rep helicase monomer bound to a partial duplex junction: single-molecule studies of fluorescently labeled enzymes. *J Mol Biol* **2004**, *336* (2), 395-408. DOI: 10.1016/j.jmb.2003.12.031.
(4) Woolley, G. A.; Lee, E. S.; Zhang, F. sGAL: a computational method for finding surface exposed sites in proteins suitable for Cys-mediated cross-linking. *Bioinformatics* **2006**, *22* (24), 3101-3102. DOI: 10.1093/bioinformatics/btl530.
(5) Kabsch, W.; Sander, C. Dictionary of protein secondary structure: pattern recognition of hydrogen-bonded and geometrical features. *Biopolymers* **1983**, *22* (12), 2577-2637. DOI: 10.1002/bip.360221211.
(6) Korolev, S.; Hsieh, J.; Gauss, G. H.; Lohman, T. M.; Waksman, G. Major domain swiveling revealed by the crystal structures of complexes of E. coli Rep helicase bound to single-stranded DNA and ADP. *Cell* **1997**, *90* (4), 635-647. DOI: 10.1016/s0092-8674(00)80525-5.
(7) Balci, H.; Arslan, S.; Myong, S.; Lohman, T. M.; Ha, T. Single-molecule nanopositioning: structural transitions of a helicase-DNA complex during ATP hydrolysis. *Biophys J* **2011**, *101* (4), 976-984. DOI: 10.1016/j.bpj.2011.07.010.
(8) *ImageJ*; U. S. National Institutes of Health: Bethesda, Maryland, USA, 1997-2018. (accessed.
(9) Suksombat, S. Binding configurations of single-stranded DNA binding protein and their influence on DNA recombinase. University of Illinois at Urbana-Champaign, ProQuest, 2015.
